# Household Laundry Appliance Resource Consumption and Operation Dataset

**DOI:** 10.1038/s41597-026-07361-6

**Published:** 2026-05-16

**Authors:** Žygimantas Jasiūnas, João Braz Ferreira, Tiago Julião, José Cecílio, Guilherme Carrilho da Graça, Pedro M. Ferreira

**Affiliations:** 1 LASIGE, Faculty of Sciences, University of Lisbon, Lisbon, Portugal; 2 Instituto Dom Luiz, Faculty of Sciences, University of Lisbon, Lisbon, Portugal

## Abstract

Household appliances, particularly washing machines and tumble dryers, account for a significant share of residential energy consumption. While energy labelling aims to encourage the adoption of more efficient appliances, actual energy and water usage are highly influenced by user behaviour and operating conditions. The user behaviour is often overlooked in existing datasets. To address this gap, the household Laundry Appliance Resource Consumption and Operation dataset (LARCO) is presented: a comprehensive, open-access dataset capturing detailed operational and environmental data from washing machines and dryers under laboratory and household conditions. The dataset includes multivariate time-series recordings at 1 Hz (and higher for vibrations and audio), covering a range of load sizes, programme settings, and ambient conditions. Laboratory data provide ground-truth measurements under controlled conditions, while household data capture real-world usage variability. Each sample includes energy, water, temperature, and humidity measurements, associated with the corresponding metadata. LARCO enables studies on appliance efficiency, user behaviour modelling, and personalised energy feedback. It supports the development of more effective strategies to bridge the gap between labelled and actual appliance performance, promoting more sustainable energy use.

## Background & Summary

The continuous growth in global population and economic development has led to a steady rise in household energy consumption, which accounts for 27% of global energy demand and significantly contributes to climate change through CO_2_ emissions^1^.

To address this challenge, energy efficiency has become a central focus of international efforts, including the European Union’s energy efficiency targets and the United Nations Sustainable Development Goals. These initiatives are designed to reduce resource consumption, promote the adoption of sustainable energy, and enhance overall energy efficiency^2,3^.

A substantial portion of residential energy use is attributed to household appliances, particularly laundry appliances such as washing machines and tumble dryers, which account for 5–11% of annual household energy consumption^4,5^. In this context, the EU has implemented energy certification and labelling schemes in their third generation to guide consumers toward more energy-efficient appliances^6^. However, while these labels provide valuable information, they do not account for the critical role of user behaviour in determining actual energy and water consumption. Real-life factors such as laundry load size, washing temperature, and cycle selection significantly influence appliance efficiency, often leading to suboptimal usage patterns and unrealised energy savings^7,8^. For instance, adopting simple practices like using lower temperature settings or half-load cycles when appropriate can reduce energy consumption by up to 30%^7^. However, without personalised, evidence-based recommendations tailored to individual usage habits, consumers remain unaware of their choices’ potential savings and environmental impact. This gap highlights the need for a deeper understanding of user behaviour and its influence on appliance efficiency.

Publicly available appliance monitoring datasets primarily focus on general household energy use, supporting tasks such as non-intrusive load monitoring (NILM) for appliance-level disaggregation^9–18^, transient analysis^19^, load analysis^20^, intrusive and non intrusive appliance classification benchmarking^13,21–23^, occupancy detection^9,14^, and grid energy demand management^18,24–26^. Supplementary Table 1 provides an exhaustive overview of publicly available appliance-monitoring datasets and compares them with the LARCO dataset. However, most of the datasets surveyed offer limited environmental and operational detail. They typically lack user-specific operational variables such as laundry load characteristics, wash cycle settings, or appliance states throughout a program, limiting their usefulness for studying the performance of wet appliances (e.g., washing machines and dryers) under real-life conditions or for developing personalised energy-efficiency feedback. These gaps motivated the creation of the LARCO dataset.

The dataset was collected within the scope of the European H2020 SATO^27^ project (SATO project, European Commission, Grant agreement ID 957128; https://cordis.europa.eu/project/id/95712), to support appliance energy efficiency research activities. It is organised into three observation categories: exploratory, laboratory, and household. The exploratory data comprise the initial recordings collected for appliance efficiency research, focusing primarily on energy and water consumption during cotton programmes with varying load weights. The laboratory data samples were acquired systematically under controlled ambient temperature conditions, including detailed sensor measurements to provide precise ground truth for appliance behaviour. In contrast, the household data reflect real-world appliance usage in domestic settings, capturing natural variability in user behaviour and operating conditions. While cotton programmes are the primary focus – being among the most commonly used and employed in energy labelling methodologies – the dataset also includes other washing programmes to enable a more comprehensive analysis of appliance performance.

Each sample in the dataset contains multivariate time-series data, capturing energy consumption, operational parameters such as water consumption, operational temperatures, and environmental factors including ambient temperature and water pressure, across a range of load sizes and water heating temperatures.

This detailed approach to data collection allows for a deeper understanding of how environmental factors, load size, and programme settings influence appliance performance. It enables studies on appliance assessment, optimisation, and the development of more accurate, personalised energy-efficiency recommendations. This work bridges the gap between theoretical energy savings and measuring practical, user-driven outcomes, ultimately promoting more sustainable household energy use.

Three application use cases are listed, which highlight the practical value of the LARCO dataset for data-driven energy efficiency:

- **Soft-sensor development:** One motivation for the LARCO dataset arose in the SATO project: the need to define and measure the real-life energy efficiency of washing machines. This use case has been realised through the development of a soft sensor^28^ that enables a mobile application currently under development. The application provides real-life energy performance data on users’ washing practices and efficiency recommendations tailored to their specific appliances when connected to an off-the-shelf smart power plug. More details are provided in the *Key Performance Indicators* Subsection of the *Methods* Section.
- **Real-life energy assessment:** The dataset enables the future development of concepts and methods that lead to real-life, user-driven, dynamic alternatives to the static performance indicators provided by manufacturers in European energy labels.
- **Environmental sensitivity analysis:** The dataset enables researchers to examine the impact of external factors, such as ambient temperature, humidity, and user behaviour, on the thermodynamic performance and operational energy efficiency of washing and drying appliances.

## Methods

### Description of the laboratory setting

**Fig. 1:**
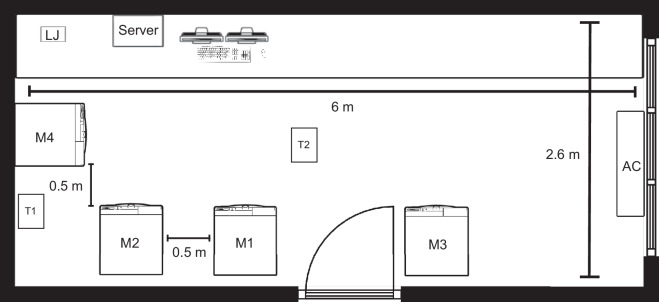
Laboratory floor plan with appliance and temperature sensor locations.

Laundry data is recorded in laboratory and household settings. As depicted in Fig. 1, the laboratory has an AC unit to control the ambient temperature during data acquisition. In the experiments, three ambient temperature settings were considered: *warm*, *neutral*, and *cold*. The ambient temperature setting in the experiment observation metadata file refers to the temperature set-point used on the AC during the experiment. Furthermore, two SHT35 sensors were placed in the laboratory to measure ambient temperature and humidity. One (T1) is placed on the opposite side of the AC near the wall. The other (T2) is placed in the centre of the laboratory. The sensors are positioned at a height equal to the centre of the drum of the laundry appliances.

The laboratory accommodates four laundry appliances simultaneously, whose stations are labelled: “M1”, “M2”, “M3”, and “M4”. The data acquisition metadata file refers to the appliance stations using these labels. M1, M2, and M4 are separated by 0.5m from one another. The data acquisition server and data acquisition boards are located in the top-left corner of the laboratory. “LJ” refers to a LabJack^29^ data acquisition board used to acquire data from the sensors.

### Laboratory data acquisition setup

This section outlines the implementation of the laundry appliance data acquisition system. It captures data from three measurement dimensions during the execution of appliance operational cycles: energy consumption, operational patterns, and environmental factors. The collected data is processed and stored using custom-developed software and a data storage system.

#### Incorporated sensors

**Fig. 2:**
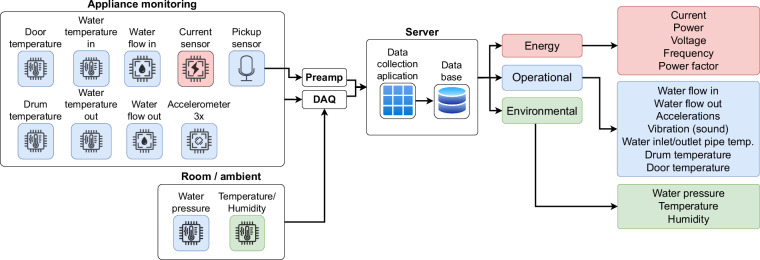
High-level appliance monitoring system architecture with the three measurement dimensions and the corresponding outputs.

The general architecture of the appliance monitoring system in the laboratory setting is illustrated in Fig. 2.

A PZEM-004T digital current sensor is used for energy dimension monitoring. Khwanrit *et al*. have shown^30^ that this sensor has a superb metering accuracy of ±0.5%. The sensor provides considerable energy features: RMS Current (*I*), RMS voltage (*V*), power (*W*), energy (*Wh*), power line frequency (Hz), and power factor (*pf*). The sensor communicates with the data acquisition board using the UART communication protocol at a rate of 9600 bit/s via the serial receiver and transmitter ports. The sensor is placed between the appliance and the wall socket, similar to a smart plug. These are the measurement ranges of the PZEM-004T sensor:

- Voltage: 80–260 V, resolution of 0.1 V.
- Current: 0–100 A, starting measure current of 0.02 A, resolution of 0.01 A.
- Active power: 0–23 kW, starting measure power of 0.4 W, resolution of 0.1 W.
- Power factor. Measuring range: 0.00–1.00, resolution 0.01.
- Frequency. Measuring range: 45Hz–65Hz, resolution 0.1 Hz.
- Active energy: 0–9999.99 kWh, resolution 1.00 Wh.

In the operational dimension, water consumption patterns for the appliances that use water are monitored using flow sensors. The inlet and outlet water patterns and volumes are measured in the washing machine. A highly accurate FS-300A G3/4 water flow sensor was selected. It is based on the Hall effect, producing square wave pulses when water passes through the sensor. Typically, pulses must be converted into litres per minute or millilitres per second. These are the technical features of the FS300A G3/4 flow-meter:

- Flow range: 1 L min^−1^ to 60 L min^−1^
- Working pressure: < 2 MPa
- Operating temperature: ≤ 80 °C
- Liquid temperature: ≤ 120 °C

Complex appliances such as washing machines heat the water inside the tub. Hence, the tub temperature is monitored to capture the heating patterns and temperatures of washing cycles. For washing machines, DS18B20 digital waterproof temperature sensors are used to measure the temperature of the inlet valve, drain hose, and bottom of the tub. They are also attached to the outside of the washing machine drum door. For tumble dryers, the sensor is placed inside the lint trap chamber. The DS18B20 sensor supports 1-wire communication, meaning multiple sensors can be connected under the same communication bus. Based on the manufacturer-provided technical sheet, the temperature measuring range is −55 °C to +125 °C, with an accuracy of ±0.5 °C.

Appliances such as washing machines, dishwashers, and tumble dryers have rotary parts that cause machines to vibrate. Accelerometers can capture this data, potentially revealing load, rotation speeds, and faults. Hence, vibration data is rich in various information about the operational state of the appliance. The ADXL-345 3-axis digital accelerometer sensor was used to monitor vibrations. It has a full-scale programmable resolution range from ±2 g to ±16 g. In the laboratory, three sensors are attached to washing machines, on the top, left side, and back panels. The sensor is digital and communicates using the I2C bus protocol. It supplies acceleration data along the X, Y, and Z axes. Additionally, a piezo AKG C411 vibration pickup sensor is used to measure vibrations.

For the environmental dimension, ambient temperature, humidity, and water pressure were considered. An SHT-35 digital temperature/humidity sensor is incorporated into the monitoring solution to monitor ambient temperature. The sensor is fully calibrated, linearised, and temperature-compensated, producing a digital output. It communicates via the I2C bus protocol, having an accuracy of ±1.5% relative humidity (RH) and ±0.2 °C ambient temperature. Its temperature measuring range is from −40 °C to 90 °C. Furthermore, water pressure is measured using a Gravity analogue water pressure sensor (AWPS). It has an accuracy of 0.5%–1% of full scale (FS). Within the operational temperature range of 0–55 °C, the typical accuracy is 0.5% FS. The sensor outputs a voltage converted into water pressure measured in megapascal (MPa).

A summary of the sensors used and their data sampling rates can be consulted in Table 1. It is worth noting that not all sensors are used in the household data acquisition setting. For example, a pickup microphone sensor is not used due to privacy issues.

**Table 1:** Summary of sensors used in the laboratory data acquisition system.

Sensor type	Sensor model	Derived quantity	Units	Communication	Sampling frequency
**Energy**					
Current Sensor	PZEM-004T	Power	W	Serial (UART)	1 Hz
		Current	A		
		Voltage	V		
		Power Factor	PF (*ϕ*)		
		Frequency	Hz		
**Operational**					
Water Flow Sensor	FS-300A	Flow Rate	L s^−1^	Pulse Output	1 Hz
Water Volume Meter		Volume	mL		
Temperature Sensor	DHT22	Temperature	^∘^C	One-Wire	1 Hz
Accelerometer	ADXL-345	Acceleration	g	I2C	200 Hz
Vibration Pickup	AKG C411	Vibration	mV	Analog	11 kHz
**Environment**					
Temp./Humidity	SHT35	Temperature	^∘^C	I2C	1 Hz
		Relative Humidity	%		
Water Pressure Sensor	Gravity AWPS	Pressure	MPa	Analog	1 Hz

#### Sensor calibration

Most sensors employed in this investigation are digital and arrive pre-calibrated from the manufacturer. However, calibration is essential for water pressure and water flow sensors. The water flow sensor operates by rotating a magnetised pin-wheel as water flows through it, generating square wave pulses. These pulses are registered by LabJack’s internal digital registers, which can then be accessed via request-response functions by programs written in the C programming language, using LabJack C libraries. The following formula calculates the flow rate:

1 $${\rm{Flow}}\,{\rm{rate}}={\rm{Pulses}}\times {\rm{Calibration}}\,{\rm{factor}}\quad [\frac{{\rm{L}}}{\min }]$$

The calibration factor, expressed in litres per pulse, was determined by pouring measured quantities of water through the sensor and comparing the ground truth value to the measurement.

The analogue water pressure sensor operates with a standard 5 V voltage input and delivers a linear voltage output ranging from 0.5 V to 4.5 V, corresponding to a pressure range of 0 to 1.6 MPa.

The sensor was supplied with a 5 V power source to improve measurement accuracy. Its output at 0 kPa was measured using an oscilloscope, revealing a slightly elevated offset of 0.6 V instead of the nominal 0.5 V stated in the datasheet^31^. Incorporating this offset into the calibration enhances the reliability and precision of the pressure readings. Therefore, the pressure *P* is computed using a linear calibration formula derived from the sensor’s specifications and the measured offset:

$$P=\frac{1600}{4.5-0.6}\times (V\,-\,0.6)\,[{\rm{kPa}}],$$

where *V* is the measured voltage from the sensor and *P* is the resulting pressure in kilopascals. This adjusted formula ensures that the calculated pressure reflects the actual conditions more accurately by accounting for sensor-specific variations under controlled power supply conditions.

#### Sensor placement

**Fig. 3:**
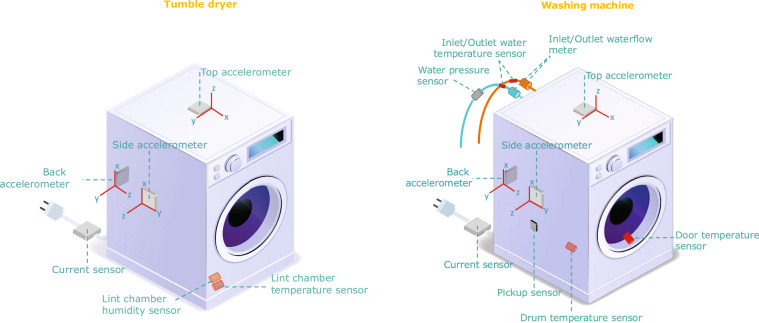
Sensor placement on the laundry appliances.

The sensor placement is illustrated in Fig. 3. For the washing machine, temperature sensors are placed at the bottom of the drum and at the drum door. Both are insulated with thermoplastic foam. As mentioned, three accelerometers are attached to the appliances, at the top, back, and side. The directions of the axes are marked in the figure. The back accelerometer is mounted in line with the centre of the drum spider on the appliance. The side accelerometer is positioned at the centre of the drum from the perspective of a front-load washing machine, and it is physically attached to the left side of the outer wall. The top-side accelerometer is positioned at the center of the washing machine. In the laboratory setting, a piezoelectric pick-up sensor is placed on the opposite side of the “side accelerometer” (only in the M1 station). Each inlet and outlet hose has a temperature sensor attached to it, as well as water flow sensors. The temperature sensors are attached to the hose pipes and insulated with thermoplastic foam. In household settings, exhaust hoses are not equipped with water flow sensors. This decision was taken by observations made during laboratory data acquisition. Due to their rotary components, Hall-effect water-flow sensors are prone to blockage by particles like small fabric strings, potentially impeding the washing machine’s water drainage. Therefore, to prevent risks such as damage to the exhaust pump, exhaust water flow sensors were not installed in household setups. The washing machine and water pressure sensor are connected to the water supply via a “T” connector.

#### Data acquisition system in the laboratory

**Fig. 4:**
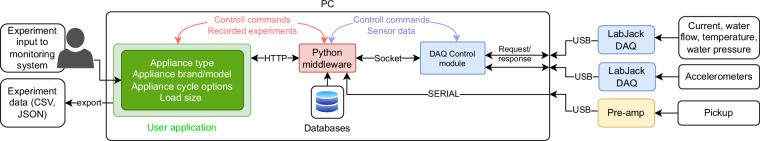
Laboratory setting data acquisition architecture.

The architecture of the laboratory data acquisition system is illustrated in Fig. 4. A user-friendly application simplifies the process of data acquisition from the appliances. It allows the definition of sample data acquisition parameters, stores the sample metadata, launches the data acquisition experiment, and allows selecting and exporting data from samples. A middleware layer written in the Python programming language coordinates the interaction between the user application, database operations, and communication with the data acquisition modules. The middleware uses two databases: MongoDB^32^ for low-frequency sensor data and InfluxDB^33^ for accelerometer data. It receives commands from the user application and forwards them to a control module written in the C programming language that communicates through USB interfaces and controls the data acquisition devices. The control module is responsible for reading sensor measurements and sending them back to the middleware for storage. The LabJack T7^29^ DAQ module is used, offering accurate and precise measurement capabilities and supporting high-speed analogue inputs and digital communication via USB or Ethernet. Two DAQs are utilised: one for slower data acquisition (1Hz) of current, water flow, temperature, and pressure sensors, and another for faster acquisition (200Hz) from accelerometers. An M-Track Duo^34^ 2-channel USB audio preamplifier board is used to sample the AKG C411 piezo pickup sensor. The audio file is stored in a lossless format (WAV).

#### Appliance cycle labelling

**Fig. 5:**
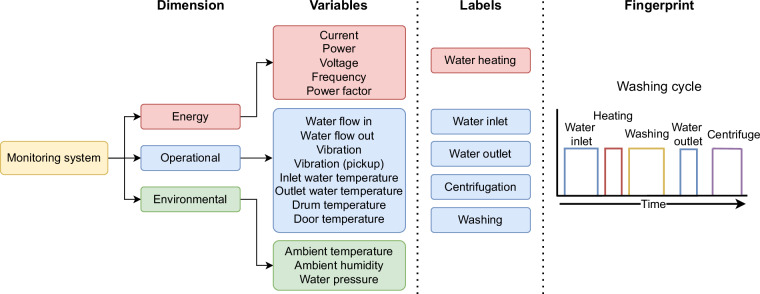
Appliance cycle labelling.

Each recorded appliance cycle in the dataset is annotated with a fingerprint, a structured representation of key functional stages and resource usage patterns. These fingerprints are constructed from multivariate time series data, encoding the temporal sequence and duration of distinct functions within each cycle. As shown in Fig. 5, the raw sensor data are labelled according to different data segments that correspond to physical events, such as water inlet/outlet, heating, washing, and centrifugation. The resulting fingerprints capture appliance-specific behaviours that differ across appliance brands and their operational setup. To generate these labels, a combination of rule-based detection and manual annotation based on sensor signals was used:

- **Heating phases:** The labelling method was identified from electrical current measurements, based on the distinct consumption of heating elements compared to the motors. During typical motor operation (e.g., drum rotation), the current averages around 2 A. In contrast, the heater activation consistently draws 7 A to 8 A. To exploit this physical distinction, a threshold of 6.5 A was applied. This value provides a robust safety margin above the motor’s peak operating current and below the heater’s minimum draw, ensuring the heating_label is reliably assigned across different machine models without requiring machine-specific tuning.
- **Water flow:**Labels were derived deterministically by thresholding the water-flow sensor signals. When either the inlet or outlet flow sensor detected movement, the system tagged the corresponding interval as water_label = 1 (inlet) or water_label = −1 (outlet). Periods with no flow activity are marked with zero.
- **Centrifugation phases:**These high-speed spinning intervals were manually annotated to ensure the highest possible label fidelity. While accelerometer data captures vibration, automated thresholding proved susceptible to false positives from environmental noise or machine imbalances. Therefore, to establish high-quality ground truth for benchmarking, manual validation was employed. This process involved visual inspection of power and current patterns combined with the accelerometer spectrogram, prioritising label accuracy over immediate scalability.
- **Washing phase:** Defined as the operational window bounded by the first water inlet event and the final centrifugation event. This implicit definition captures the full active cycle duration.

#### Household appliance data acquisition kit

**Fig. 6:**
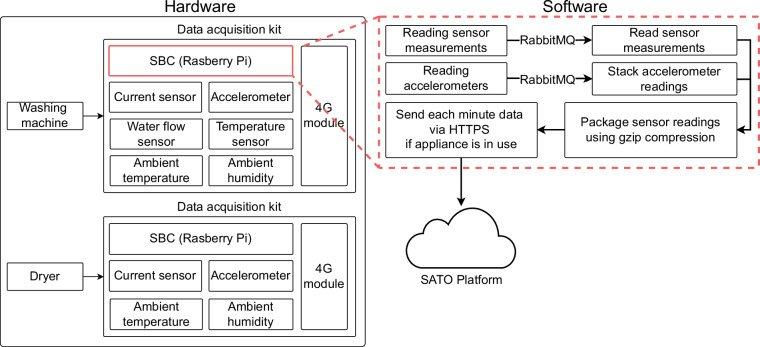
Household appliance data acquisition kit architecture.

A portable and compact data acquisition kit was developed for deployment in households. To ensure consistent data quality between the laboratory and household monitoring environments, identical sensors were used in the data acquisition kit. Figure 6 illustrates the high-level architecture of the kit.

A Raspberry Pi 4^35^ single-board computer (SBC) is used because it can handle all the required sensor communication and interfacing through its 40-pin GPIO (general-purpose input/output), all the computing processes using its 64-bit quad-core processor, and upload data through a 4G modem connected to a USB port. Moreover, it allows decreasing the size of the kit by avoiding the use of a DAQ module. The acquisition kit begins recording and storing data if the appliance changes its state from idle to active. Every minute, a process sends packaged sensor measurements data to a central SATO^27^ server using a 4G modem.

**Fig. 7:**
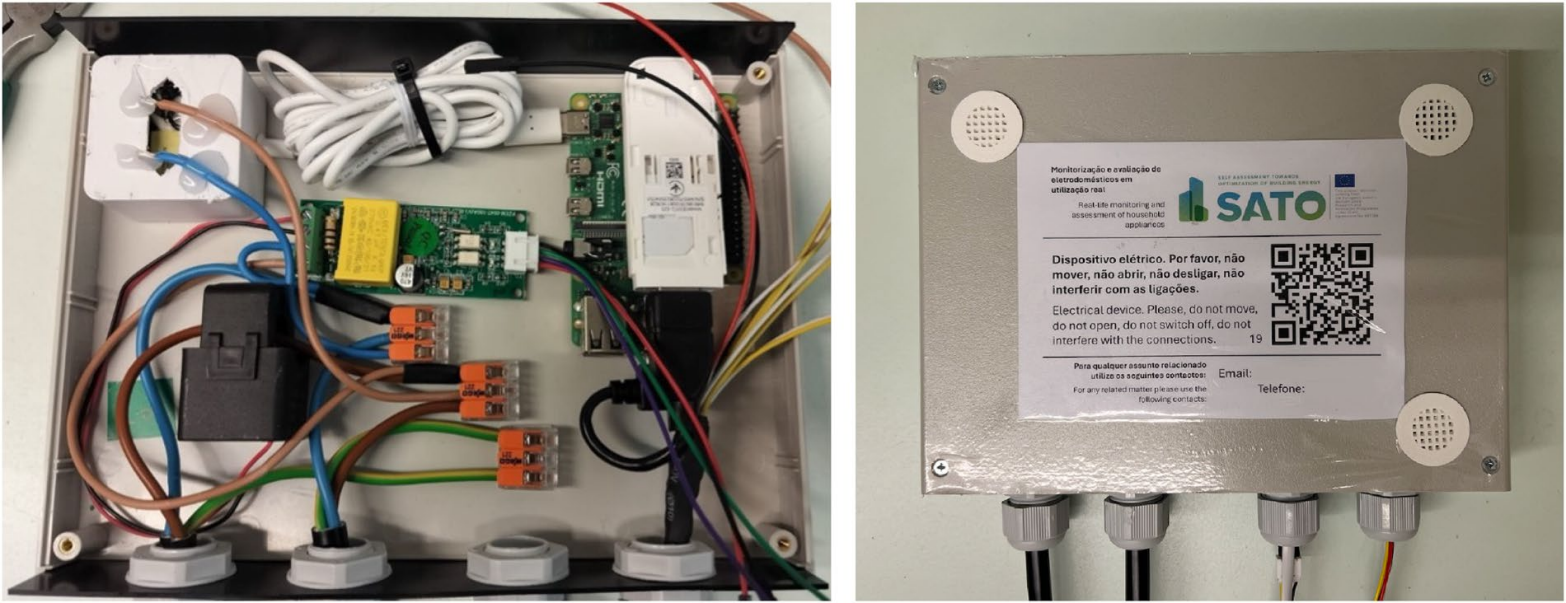
Data acquisition kit for monitoring household appliance real-life use.

Figure 7 illustrates the acquisition kits that were installed to monitor household appliances in the SATO project residential pilot (Seixal, Portugal). On the left side, the box is open, revealing all the internal components, while on the right side, the box is securely closed, with all the necessary information provided to the participant on the back of the box.

#### Systematic laboratory data acquisition protocol

A systematic data acquisition protocol was implemented for washing machines and dryers. The protocol includes combinations of washing and drying programs with varying load sizes, program temperatures, and ambient conditions. A summary of the protocol is provided in Table 2.

**Table 2:** Summary of systematic washing machine and dryer data acquisition protocol in the laboratory.

Type	Ambient temperature	Programme	Laundry load (kg)
Dryer	16 °C, 31 °C	Cotton standard	1, 2, 3, …, max
	16 °C, 31 °C	Cotton extra	
	25 °C	Other available programmes	
Washer	16 °C, 31 °C	Cotton 0, 20, 30, 40, 60, 90	0, 2, 4, …, max - 1
		ECO 40–60	
	25 °C	Other available programmes	0, 2, 4, …, max - 1
			(in some cases: zero, half, and full load)

For washing machines, data collection was conducted by running washing cycle tests with incremental laundry loads, increased by two kilograms per test, starting from an empty drum up to one kilogram below the machine’s rated capacity. For example, if the washing machine had a maximum capacity of 12 kg, the maximum load would be 11 kg. In the case of dryers, the wet laundry load was varied in 1 kg steps, ranging from 1 kg to the maximum rated capacity of the machine. In the laboratory, before loading the appliances, the laundry weight was measured as closely as possible to the target weight, within an error margin of ±50g.

The *Cotton* programs were tested at all available cycle temperatures, ranging from 0 °C (“Cotton cold”) to 90 °C (“Cotton warm”). In the laboratory, ambient temperature was regulated by an air-conditioning system, enabling *Cotton* and *ECO 40–60* programme measurements under two controlled conditions: 16 °C (“cold” ambient) and 31 °C (“warm” ambient). Additional washing programs-other than *Cotton* and *ECO 40–60* were recorded exclusively at a (“neutral” ambient) temperature of 25 °C.

For each washing and drying cycle, before and after the cycle, the laundry mass was measured and recorded with a resolution of 1 g. In addition, the mass of condensate collected in the dryer water tank was recorded after each drying cycle.

The data acquisition system was configured to collect signals from multiple sensors at different sampling frequencies, depending on the nature of the measurement. As shown in Table 1, the accelerometer data were acquired at a rate of 200 Hz and the signal from the audio pickup was sampled at 11 kHz for a richer representation of transient acoustic. All remaining energy, operational, and environmental parameters (such as power consumption, temperature, and humidity) were recorded at a sampling frequency of 1 Hz.

### Selected household appliances

The SATO project residential pilot was initiated to evaluate the real-life energy performance of households and household appliances. In this context, data acquisition kits were installed in selected households to gather detailed data on the real-life use of specific appliances. Before the deployment of the data acquisition kits, a selection was made from the available households in SATO’s residential pilot to create a pool of appliances with a diverse range of machine types, ages, and usage. Additionally, the selection focused on typical European models (front-loading with cold water supply). The selected appliances consider legacy and modern machines (years 2004 to 2020) and various load capacities (from 6 to 10 kg). Eight monitoring kits have been deployed in seven households, monitoring the real-life utilisation of front-loading washing machines and dryers. Note that due to the sample size and the focus on a single geographical area, these data are not statistically representative of the broader population. Instead, they are primarily used for exploratory analysis of detailed usage patterns.

Table 3 describes the monitored appliance’s type, brand, and model. It also includes metadata such as the machine’s energy label provided by the manufacturer, max load, and max spin speed.

**Table 3:** Summary of monitored laundry appliances in the residential pilot (the energy efficiency class of appliances with IDs 3 to 7 relates to pre March 2021 European Union regulations).

ID	Type	Brand	Model	Rated capacity	Rated spin speed	Year	Energy efficiency class
1	Washer/dryer	Hoover	WDYN654D-30S	6 kg / 5 kg	1400	2010	B
2	Washer	Samsung	WW80T554DTW/S3	8 kg	1400	2020	B
3	Washer	Electrolux	EWF1272EOW	7 kg	1200	2014	A+++
4	Washer	Whirlpool	FMG 823B	8 kg	1200	2014	A+++
5	Washer	AEG	L6FBG141	10 kg	1400	2019	A+++
6	Washer	Samsung	WF80F5E0W2W/EP	8 kg	1200	2011	A+++
6	Dryer	Beko	DHS7412PA0	7 kg	N/A	2019	A++
7	Washer	Balay	3TS866EE/23	6 kg	1000	2013	A+++

As mentioned, during the exploratory stage of the study, data records were collected from an initial group of appliances to establish the energy efficiency research methodology. These data are included in the dataset under the exploratory category. Table 4 describes the appliance characteristics, showing also the number of data acquisition experiments performed.

**Table 4:** Summary of washer appliances monitored during the exploratory stage, (the energy efficiency class of the Samsung and Siemens appliances relates to pre March 2021 European Union regulations).

Brand	Model	Rated capacity	Rated spin speed	Year	Energy efficiency class	N^*o*^ of experiments
Bosh	WFO2063EE	6 kg	1000 rpm	2002	Unknown	6
Indesit	IWC71252	7 kg	1200 rpm	2014	E	4
Samsung	WF1802WPC	8 kg	1200 rpm	2011	A+++	5
Siemens	WM12N278EP	8 kg	1200 rpm	2018	A+++	5
LG	F4WV710P1	10.5 kg	1360 rpm	2019	A	6
**Total**						**26**

### Selected laboratory appliances

Washing machines and dryers for the laboratory were provided by the Portuguese Worten^36^ company. As for households, the supply was for front-loading units with a cold water supply. All test units were new and from recent production years, with rated capacities from 6 to 12 kg. Table 5 lists the observed appliances and the corresponding number of experiments conducted. The “Becken BWM5381 IX flt” washing machine had a known drum calibration problem. The dataset also considers one unit of the same washing machine without the calibration problem. Hence, the data from these two washing machines can be used for fault analysis.

**Table 5:** Summary of appliances monitored in the laboratory.

Type	Brand	Model	Rated capacity	Rated spin speed	Year	Energy efficiency class	N° of experiments
Washer	Becken	BWM5379 IX	8 kg	1400 rpm	2018	B	79
	Becken	BWM5381 IX	12 kg	1400 rpm	2021	B	93
	Becken	BWM5381 IX flt	12 kg	1400 rpm	2021	B	106
	KUBO	KBWM8321	6 kg	1000 rpm	2019	E	92
	KUBO	KBWM8321	6 kg	1000 rpm	2019	E	93
	Kunft	WM5316	7 kg	1400 rpm	2018	E	80
	Kunft	WM5317	8 kg	1400 rpm	2018	E	100
Dryer	Becken	BDM2741 HP	8 kg	N/A	2017	A++	80
	Becken	BDM8331 HP	8 kg	N/A	2019	A++	80
**Total**							**803**

(The energy efficiency class of dryers relates to pre March 2021 European Union regulations).

### Supplementary weather data

To account for environmental conditions during the laundry processes, a supplementary file of weather data points was added to the dataset framework using the OpenWeather API^37^ (https://api.openweathermap.org). Historical data on ambient temperature, humidity, and atmospheric pressure were retrieved for the reference geographic locations of the laboratory in Lisbon and the household deployment area in Seixal, Portugal. Data collection was automated using a Python script (weather_data.ipynb) available in the GitHub repository, where the specific coordinates for both locations are documented. While the weather queries were synchronized with the initiation timestamp of each laundry cycle from aggregated_data.csv, the resulting environmental variables are stored in a standalone weather_data.csv file. This separate weather file maintains strict temporal alignment with the primary dataset through matching timestamps, ensuring that environmental conditions can be accurately mapped to each cycle.

### Key performance indicators

To demonstrate the analytical potential of the LARCO dataset, its application in assessing real-life energy efficiency is illustrated. Specifically, a Key Performance Indicator (KPI) framework, previously proposed in^28^, was applied, with two KPIs to assess the real-life efficiency of resource use: energy (*r*_*e*_) and water (*r*_*w*_), relative to the mass of laundry washed. These are the energy consumption per kilogram of laundry, defined as:

2 $$E=\frac{{r}_{e}}{m}\quad [\frac{{\rm{Wh}}}{{\rm{kg}}}],$$

and the water consumption per kilogram of laundry:

3 $$H=\frac{{r}_{w}}{m}\quad [\frac{{\rm{L}}}{{\rm{kg}}}].$$

*E* and *H* provide a quantitative measure of how efficiently a washing machine uses resources. Since the mass of the washed laundry (*m*) might not be available, a reliable estimate is essential for effective performance assessment. For that purpose, resulting from this dataset, an IoT-based soft sensor using machine learning was previously proposed^28^. To quantify the total resource consumption per cycle, the collected time-series data are integrated over the washing using the following equations:

4 $${r}_{e}=\frac{{\int }_{{t}_{0}}^{{t}_{f}}P(t)\,dt}{3600}\quad [{\rm{Wh}}]$$

5 $${r}_{w}={\int }_{{t}_{0}}^{{t}_{f}}\frac{\mathop{V}\limits^{.}(t)}{60}\,dt\quad [{\rm{L}}]$$

*P*(*t*) denotes instantaneous power (W), sampled every second. The integral is scaled by 1/3600 to yield energy in watt-hour (Wh). Likewise, $\mathop{V}\limits^{.}(t)$ is the water flow rate in litres per minute, integrated over time and scaled by 1/60 to produce the total litres.

The *E* and *H* KPIs can be combined for economic assessment by multiplying each by the respective cost (e.g., price per kWh or litre of water). The clean water supply energy expenditure can be incorporated into a unified energy KPI for a more holistic view of energy use performance. This KPI, denoted *E*_*n*_, utilizes the water-energy nexus concept^38^, which states that the production of clean water incurs energy costs due to pumping, purification, and distribution. Considering that in the European context this cost is on average 0.78 Wh/L^38^, the combined energy use KPI is expressed as:

6 $${E}_{n}=E+0.78\times H\quad [\frac{{\rm{Wh}}}{{\rm{kg}}}]$$

The *E*, *H*, and *E*_*n*_ KPIs form a comprehensive framework for assessing the energy use and resource efficiency of washing machines. *E*_*n*_ in particular offers a more comprehensive perspective by accounting for direct and indirect energy consumption, supporting informed decisions in the design, operation, and optimization of washing systems. The same principle applies to the drying machine, considering a KPI similar to *E*, which expresses the energy consumed per kilogram of laundry load dried.

In typical domestic settings, the mass of the washed laundry (*m*) is not available, which introduces uncertainty if users rely on inaccurate estimates. However, this limitation highlights one specific value of the LARCO dataset: by providing ground-truth mass measurements alongside operational signals in the laboratory data, it enables the development and validation of “soft sensors” using, for instance, machine learning models^28^ capable of estimating load size, thereby reducing the uncertainty associated with the data. Thus, while the KPIs depend on load data, this dataset provides the necessary ground truth to bridge the gap between theoretical KPIs and real-world applications.

## Data Records

The dataset is available at the Zenodo repository under the title ’Household Laundry Appliance Resource Consumption and Operation Dataset’^39^.

**Fig. 8:**
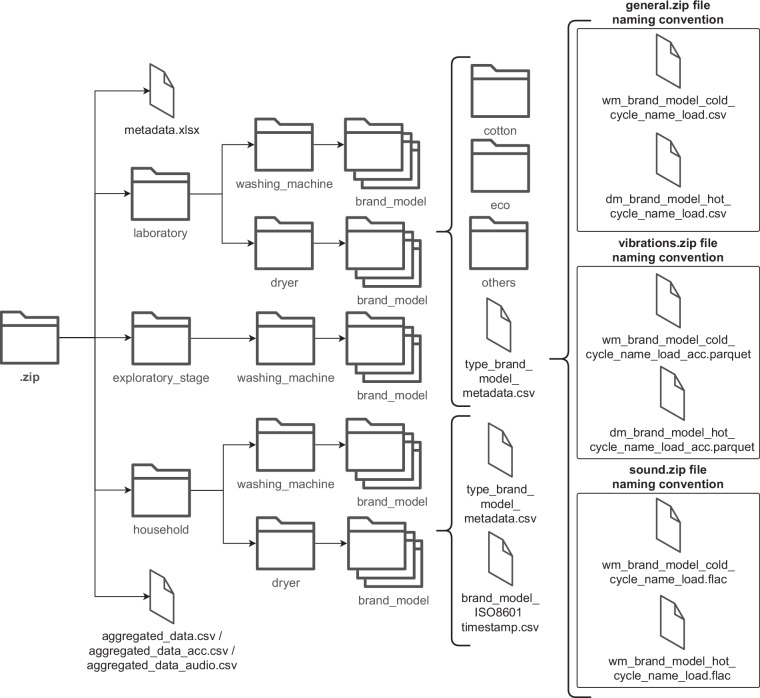
Overview of the dataset ZIP file structure, including record naming.

The deposit is organised in three top-level ZIP files: general.zip (energy, operational, and environmental data, acquired at 1 Hz), vibrations.zip (triaxial accelerometers data, acquired at 200 Hz), and audio.zip (pickup sensor audio data, acquired at 11025 Hz in WAV format that was then compressed to FLAC). Each file follows the folder hierarchy illustrated in Fig. 8. The general.zip file contains three folders: laboratory, exploratory_stage, and household. Since vibrations and sound were not recorded during the exploratory stage, the exploratory_stage folder is not included in vibrations.zip and audio.zip. Due to privacy concerns, sound recordings were only made in laboratory experiments. Therefore, the audio.zip file contains only the laboratory folder, which includes data recorded at the M1 station. Inside the washing_machine and dryer directories, each device has its own folder, with cycles organised into subfolders: cotton, eco, and others.

Laboratory and experimental data files follow a common naming convention: <appliance>_<brand>_<model>_<ambient>_<cycle_name>_<load>, where <appliance> is wm (washing machine) or dr (dryer), <brand>_<model> denotes the appliance make and model, <ambient> is hot, warm or cold (hot ≈ 31 °C, warm ≈ 25 °C, cold ≈ 16 °C), <cycle_name> is the program used, and <load> is the target laundry mass, rounded to the nearest integer. For household appliances, the acquired sample naming convention follows the format: <appliance>_<brand>_<model>_<ISO8601-timestamp>.

### Metadada

An accompanying metadata.xlsx file outlines the variables measured, including the sensor type, recording environment, unit of measurement, and sampling frequency. Each entry briefly describes the variable’s role within the dataset, ensuring clarity and consistency in how the data are interpreted and used. The type_brand_model_metadata.csv file contains detailed metadata for each appliance, including its type, brand, model, year of release, and energy efficiency label. It also specifies the environment in which the appliance was recorded (e.g., laboratory or household) and includes a station ID for samples recorded in the laboratory. When available, appliance energy performance data is sourced from the European Product Registry for Energy Labelling (EPREL) database^40^. If the information was unavailable in EPREL, it was collected from official manufacturer booklets and product web pages.

### Aggregated data

Each ZIP file contains a CSV file named aggregated_data.csv, providing all essential information about each experiment. Additionally, specific datasets are provided for different types of data: operational data is stored in aggregated_data.csv, accelerometer data in aggregated_data_acc.csv, and audio data in aggregated_data_audio.csv. These files include the file path and the data acquisition location (laboratory, household, or exploratory). For experiments conducted in the laboratory, additional information is provided, including the ground truth of the laundry weight before and after washing or drying. These weight measurements are recorded in kilograms with a precision of three decimal places. For dryer experiments, the water quantity is also recorded and measured in litres with the same level of precision. Additionally, the file includes statistical properties derived from the general data samples. A comprehensive list of aggregated fields for each experiment is provided in Table 6 for aggregated_data.csv, and in Table 7 for aggregated_data_acc.csv and aggregated_data_audio.csv. In addition to the listing in Table 6, the file aggregated_data.csv contains the mean, median, variance, maximum, minimum, range, and standard deviation statistical measures of each experiment, computed for the sensor measurements “power”, “current”, “frequency”, “power_factor”, “voltage”, “drum_door_temp”, “drum_temperature”, “water_temperature_in”, “water_temperature_out”, “ambient_temperature_1”, “ambient_temperature_2”, “ambient_humidity_1”, “ambient_humidity_2”, “pressure”, “inside_temperature”, “inside_temperature_2”, and “inside_humidity”.

**Table 6:** Column definitions from aggregated_data.csv.

Field	Column name	Description
Timestamp	timestamp	Timestamp of cycle initiation in ISO 8601 format.
Cycle ID	unique_monitoring_id	Unique identifier linking general, accelerometer and audio data
File path	file_path	Path to the cycle file relative to aggregated_data.csv.
File name	file_name	Cycle file name.
Appliance type	type	Shorthand appliance type: wm = washing machine, dr = dryer.
Brand	brand	Appliance brand.
Model	model	Appliance model.
State	state	Appliance condition: new, used or faulty.
Machine	machine	Brand and model combined using underscore.
Program	program	Operational cycle name.
Program heat	program_heat	Predefined heat level for the program.
Revolutions per minute	rpm	Revolutions per minute (RPM) for the cycle. Applicable to washing machines.
Environment	environment	Acquisition setting: laboratory, household, or experimental.
Ambient temperature	ambient_temp	AC temperature setting in lab recordings.
AC setting	ac_set	Possible values: hot = 31 ^∘^C, warm = 25 ^∘^C, cold = 16 ^∘^C.
Target load weight	target_load	Discrete value of desired load weight (kg).
Actual load weight	actual_weight	Measured dry load weight (kg), accurate to 0.001.
Load weight after cycle	post_cycle_weight	Measured wet load weight (kg), accurate to 0.001.
Water in the tank	water_tank_quantity	Water collected in dryer tank (L), accurate to 0.001.
Total energy consumed	energy_consumed	Total energy used (kWh) during the cycle.
Total water consumed	water_consumed	Total water used (L), applicable for washing machines.
Ground truth water KPI	w_kpi	Water consumption per kg of load.
Ground truth energy KPI	e_kpi	Energy consumption per kg of load.
Ground truth energy nexus KPI	ew_kpi	Combined energy and water KPI (accumulated).
Cycle duration	cycle_duration	Cycle length in minutes.
Has accelerometer	has_accelerometer	Boolean flag: 1 = accelerometer data available.
Has audio	has_audio	Boolean flag: 1 = audio data available.

**Table 7:** Summary of aggregated_data_acc.csv columns.

Field	Column name	Description
Timestamp	timestamp	Timestamp of cycle initiation, expressed in ISO 8601 format.
Cycle ID	unique_monitoring_id	A unique identifier to link operational, accelerometer, and audio data.
File path	file_path	Path to the cycle file relative to the aggregated_data.csv file.
File name	file_name	Cycle file name.
Energy performance file	ep_file_path	Relative path to energy performance file.
Appliance type	type	Appliance class: washing machine, dryer.
Type	type	Represents the type of appliance.
Brand	brand	Appliance brand.
Model	model	Appliance model.
State	state	Appliance condition. Possible values include: new, faulty.
Machine	machine	Combination of appliance brand and model, separated by an underscore.
Program	program	Appliance operational cycle name.
Program heat	program_heat	Known or predefined program heat level.
Revolutions per minute	rmp	Revolutions per minute (RPM) for the selected cycle.
Cycle	cycle	Combination of program name and heat setting, separated by an underscore.
Environment	environment	Location of data acquisition: laboratory, household, or experimental.
Ambient temperature	ambient_temp	For lab environments, this is the AC temperature setting.
AC setting	ac_set	Possible values: hot = 31 °C, warm = 25 °C, cold = 16 °C.
Target load weight	target_load	Actual load weight (kg), rounded to a discrete value.
Actual load weight	actual_weight	Measured load weight in kg (accurate to 0.001 kg) before the cycle.
Load weight after cycle	post_cycle_weight	Wet load weight in kg (accurate to 0.001 kg) after the cycle.
Water in the tank	water_tank_quantity	Dryer tank water volume in liters (accurate to 0.001 L). Applies to heat pump dryers.
Cycle duration	cycle_duration	Cycle duration in minutes.
Position ID	position_id	Position of the appliance (refer to lab layout/map).
Contains missing values	has_missing_values	Flags files with missing data or inconsistent acquisition frequency.

### General data

Table 8 details the data, units, and brief description for the measurements available in the general samples, grouped by the energy, environmental, and operational data categories. Each sample is a CSV file with records sampled at 1 Hz, containing the timestamp and corresponding measurements columns.

**Table 8:** Overview of features available based on the environment and appliance type.

Data	App.	Env.	Measurement	Column name	Unit	Description
Energy use	wm/dr	L / H	Voltage	voltage	V	
			Current	current	A	
			Power	power	W	
			Power factor	power_factor	pf	
			Frequency	frequency	Hz	
Indoor environment	N/A	L / H	Temperature	ambient_temperature_1	°C	Temperature from sensor 1
		L	Temperature	ambient_temperature_2	°C	Temperature from sensor 2
		L / H	Rel. Humidity	humidity_1	%	Relative humidity from sensor T1
		L	Rel. Humidity	humidity_2	%	Relative humidity from sensor T2
Water inlet	wm	L / H	Volume	water_inlet	mL	Inlet hose monitoring
Water outlet	wm	L	Volume	water_outlet	mL	Outlet hose monitoring
Water temperature in	wm	L / H	Temperature	water_temperature_in	°C	Inlet hose temperature monitoring
Water temperature out	wm	L / H	Temperature	water_temperature_out	°C	Outlet hose temperature monitoring
Water pressure	wm	L / H	Pressure	water_pressure	psi	Water inlet pressure monitoring
drum temperature	wm	L	Temperature	drum_temperature	°C	Sensor attached to bottom of drum
Door temperature	wm	L	Temperature	door_temperature	°C	Sensor attached to washing machine door
Lint trap temperature	dr	L	Temperature	inside_temperature	°C	Sensor inside lint trap chamber
Lint trap temperature 2	dr	L	Temperature	inside_temperature_2	°C	Sensor inside lint trap chamber
Lint trap humidity data	dr	L	Rel. Humidity	inside_humidity	%	Sensor inside lint trap chamber
Generated fingerprint	wm	L	Water flow activity	water_label	N/A	1 = flow in, −1 = flow out, 0 = no flow
			Heating activity	heating_label		1 = heating, 0 = not heating
			Centrifugation	centrifuge_label		1 = centrifuging, 0 = not centrifuging

App. = appliance, Env. = environment, L = laboratory, H = household, wm = washing machine, dr = dryer.

Experimental general data contains only the water inlet rolling sum in litres and power (W) measurements. The general, vibration, and audio data are linked by a shared unique_monitoring_id that can be found in the aggregated_data.csv, aggregated_data_acc.csv, and aggregated_data_audio.csv files.

### Vibration data

Table 9 describes the data contained in the accelerometer samples. As shown in Fig. 3, each appliance is instrumented with three tri-axial accelerometers mounted on the top, side, and back panels, each recording acceleration along the X, Y, and Z axes. Each sample experiment is stored in Apache Parquet^41^ format.

**Table 9:** Summary of acceleration data file. Acceleration values are expressed in m/s^2^.

Data	Appliance	Environment	Measurement	Column name	Description
Accelerometer data	wm/dr	Laboratory / Household	Vibration	top_x	Accelerometer placed on top
				top_y	
				top_z	
				side_x	Accelerometer placed on the left side
				side_y	
				side_z	
				back_x	Accelerometer placed on the back side
				back_y	
				back_z	

### Audio data

Audio was captured from the pickup sensor as an uncompressed single-channel WAV file at 11025 Hz. File names follow the same convention as the general and accelerometer data. To reduce storage without altering the signal, all recordings were transcoded losslessly to the FLAC format^42^ and stored as .flac files. No resampling, filtering, or level adjustments were applied.

#### Exploratory stage

The dataset’s structure, which lacks a timestamp, prevents rigorous checking for gaps in sequential records. Hence, the work proceeded under the assumption that no rows were missing from the data collection. A direct inspection of all features confirms zero occurrences of NaN or null values.

## Data Overview

A summary of the ZIP files content is given in Table 10. To complement the archive-level counts, Table 11 provides details on the number of recorded cycles per brand, model, type, and environment, along with program-level details for cotton, eco, and others. The last column presents the total number of cycles for a given device.

**Table 10:** Archive inventory.

ZIP	# experiments	# metadata files	Size (GB)
general.zip	1067	22	0.19
vibrations.zip	990	15	13.52
audio.zip	325	8	24.45

**Table 11:** Dataset summary of washing machines and dryers by brand, model, type, and environment.

Brand	Model	Type	Environment	General	Vibration	Audio	Cotton	Eco	Others	Total
Indensit	IWC71252	wm	exploratory	4	0	0	4	0	0	4
Samsung	WF1802WPC	wm	exploratory	5	0	0	5	0	0	5
Siemens	WM12N278EP	wm	exploratory	5	0	0	5	0	0	5
Bosh	WFO2063EE	wm	exploratory	6	0	0	6	0	0	6
Lg	F4WV710P1	wm	exploratory	6	0	0	6	0	0	6
Balay	3TS866EE	wm	household	13	7	0	0	0	13	20
Samsung	WW80T554DTW	wm	household	11	11	0	0	0	11	22
Samsung	WF80F5E0W2W	wm	household	20	6	0	0	0	20	26
Aeg	L6FBG141	wm	household	16	16	0	0	0	16	32
Whirlpool	FMG823B	wm	household	22	22	0	0	0	22	44
Hoover	WDYN654D-30S	wm	household	66	48	0	0	0	66	114
Electrolux	EWF1272EOW	wm	household	72	65	0	0	0	72	137
Becken	BWM5379IX	wm	laboratory	79	78	0	39	10	30	157
Kubo-2	KBWM8321	wm	laboratory	93	91	0	47	8	38	184
Kunft	KWM5317	wm	laboratory	100	100	0	40	10	50	200
Kunft	KWM5316	wm	laboratory	80	80	76	32	8	40	236
Becken	BWM5381IX	wm	laboratory	93	93	57	56	14	23	243
Kubo-1	KBWM8321	wm	laboratory	92	89	86	44	8	40	267
Becken-flt	BWM5381IX	wm	laboratory	106	106	106	56	14	36	318
Beko	DHS7412PA0	dr	household	18	18	0	0	0	18	36
Becken	BDM2741	dr	laboratory	80	80	0	32	0	48	160
Becken	BDM8331	dr	laboratory	80	80	0	32	0	48	160
			**Total**	**1067**	**990**	**325**	**404**	**72**	**591**	**2382**

*Type*: wm = washing machine, dr = dryer. **General** counts experiments with energy, operational, and environmental data stored in general.zip. **Vibrations** and **audio** count files recorded in parallel to the general and are stored in vibrations.zip and audio.zip.

## Technical Validation

To validate the data collected in household and experimental settings, comparative tests were performed against a laboratory-grade setup. These evaluations confirmed consistent sensor readings and acquisition fidelity across environments, supporting the overall reliability of the dataset.

Energy and water consumption values were validated through multiple approaches. First, the recorded values were compared against appliance specifications provided by the manufacturers. Additionally, energy and water consumption metrics were recalculated in accordance with European energy efficiency regulations, allowing for verification of the reported energy labels. Grid energy measurements and household water pressure readings were evaluated against national regulatory standards. Water heating profiles were cross-checked against programmed temperature settings on the appliances, while inlet water temperature readings were correlated with ambient outdoor temperatures to confirm the plausibility of the sensor readings. Laboratory ambient temperature sensors were validated by comparing the ambient temperature readings and the setpoints of the air-conditioning system. Finally, acoustic and vibration data were validated by extracting dominant frequency components and comparing them to the expected harmonics derived from the appliance spin speeds during centrifugation cycles. Together, these validation procedures establish confidence in the accuracy and representativeness of the collected data.

### Missing data

No imputation or data correction has been applied to maintain transparency and preserve the integrity of the original records. The dataset is provided in its raw form. Users are encouraged to apply domain-appropriate methods to handle missing values based on their analytical goals.

#### Laboratory

Missing data in the laboratory dataset primarily comes from two sources: (i) sensor unavailability during certain experimental runs, and (ii) technical issues encountered during acquisition. For example, measurements from the washing machine’s drainage outlet are absent in some cycles due to residue accumulation in the pinwheel mechanism of the water flow sensor. This obstruction prevented the sensor from registering pulses, resulting in missing outflow water measurements. Incomplete or inconsistent accelerometer data were also observed, likely caused by communication bottlenecks during acquisition. Such interruptions may have arisen from queue buildup in the Python middleware socket or latency in draining data from the DAQ control module (see Fig. 4), leading to temporary data dropout. Audio data from the pickup sensor was recorded only at station “M1”. Consequently, 49% of all laboratory washing machine cycles do not include audio measurements. Additionally, some data — such as drum door temperature, drum temperature, water flow temperature, accelerometer readings, and water pressure — are absent in specific samples because the sensors were not yet fully integrated into the acquisition system at the time of recording. In these cases, the affected columns within a cycle are populated with a value of −1. For washing machine data, these ‘structural’ gaps may occur as entire columns missing from the main cycle record, or as missing data files for audio and/or accelerometer measurements. Table 12 reports missing values across the 643 laboratory washing machine cycles acquired. For the dryers data (160 cycles), no files were missing.

**Table 12:** Summary of missing features (absent from the cycle) across laboratory washing machine cycle samples (*N* = 643).

Missing feature	Samples with missing data	Missing (%)
audio_samples	318	49.00
drum_temperature	89	13.84
drum_door_temp	57	8.86
water_outlet	55	8.55
pressure	7	1.09
accelerometer	6	0.93
ambient_temperature_1	6	0.93
ambient_temperature_2	6	0.93
ambient_humidity_1	6	0.93
ambient_humidity_2	6	0.93
water_temperature_out	4	0.62
water_inlet	2	0.31
water_temperature_in	2	0.31
power_factor	0	0.00
voltage	0	0.00
power	0	0.00
current	0	0.00
frequency	0	0.00

In addition to structural omissions, some files contain gaps with missing values within otherwise consecutive measurements. The within-file NaN values can arise for two main reasons:

- **Sensor interference** — occasional invalid readings, likely due to electromagnetic interference or signal attenuation in the cables connecting sensors to the acquisition system.
- **Time drift** — cases where two samples are recorded within the same second (see the “timestamp” row in the tables), typically caused by occasional slower sensor responses, resulting in one sample being redundant. While these duplicate samples are retained in the dataset, they are treated as indicators of timing inconsistencies and considered analogous to missing data in the analysis of the washing machine and dryer samples.

Table 13 details within-file missing values for the washers. For dryers, all missing entries are attributed to time drift, resulting in 0.08% missing samples (788 out of 1027900 expected samples).

**Table 13:** Summary of missing data points per recorded feature across laboratory washing cycles.

Missing feature	Expected samples	Samples with missing data	Missing (%)
ambient_humidity_1	5 183 451	3867	0.07
ambient_humidity_2	5 183 451	3867	0.07
ambient_temperature_1	5 183 451	3867	0.07
ambient_temperature_2	5 183 451	3867	0.07
current	5 242 057	9549	0.18
drum_door_temp	4 766 192	10 995	0.23
drum_temperature	4 501 745	7763	0.17
frequency	5 242 057	9549	0.18
power	5 242 057	9549	0.18
power_factor	5 242 057	9549	0.18
pressure	5 172 795	3866	0.07
timestamp	5 242 057	3977	0.08
voltage	5 242 057	9549	0.18
water_inlet	5 242 057	3977	0.08
water_outlet	5 242 057	14 858	0.28
water_temperature_in	5 220 736	17 400	0.33
water_temperature_out	5 200 421	4461	0.09
**Total**	95 276 137	147 037	0.15

Cycles affected by missing or inconsistent data, particularly for the outflow water sensor and accelerometer, are explicitly flagged in aggregated_data.csv and aggregated_data_acc.csv. The flags enable downstream users to identify, filter, or impute incomplete records as appropriate for their analyses.

Three triaxial accelerometer sensors (top, side, and back) were recorded at a sampling rate of 200 Hz. During data acquisition, the system occasionally experienced data loss, resulting in the simultaneous loss of samples across all axes. The number of missing samples for all axes is 96436442, corresponding to 7.87% of the expected measurements.

#### Household

In the household pilot, missing data within existing recordings of the general data accounted for 12.88% of the expected samples, corresponding to 202 063 measurements absent out of a total of 1568 688 expected. These losses occur despite the presence of the corresponding files and are primarily attributed to intermittent network communication failures during data acquisition, which result in partial dropouts rather than entire file loss. As with the full dropouts, the missing intervals affect all recorded variables simultaneously, indicating that the loss mechanism is system-wide rather than feature-specific. Table 14 summarises the missing data per appliance. In addition to these within-file dropouts, some experiments are missing their entire accelerometer file. Table 15 summarises the completeness of accelerometer recordings for each monitored appliance, showing the number of expected files, how many were found with valid data, and the percentage of completely missing accelerometer datasets. Table 16 presents the within-file completeness of accelerometer recordings for each monitored laundry appliance.

**Table 14:** Summary of missing operational household data due to full acquisition dropouts for each monitored laundry appliance, assuming one row per second.

ID	Type	Brand	Model	Expected rows	Actual rows	Missing rows	Missing (%)
1	Wm/Dr	Hoover	WDYN654D-30S	278146	228259	49887	17.94
2	Wm	Samsung	WW80T554DTW	39602	38869	733	1.85
3	Wm	Electrolux	EWF1272EOW	569941	566029	3912	0.69
4	Wm	Whirlpool	FMG823B	178893	169478	9415	5.26
5	Wm	AEG	L6FBG141	44702	42821	1881	4.21
6	Dr	Beko	DHS7412PA0	282050	153703	128347	45.51
6	Wm	Samsung	WF80F5E0W2W	108344	101433	6911	6.38
7	Wm	Balay	3TS866EE	67010	66033	977	1.46

*Expected rows* are computed from each file’s duration. *Actual rows* are the number of unique seconds present. *Missing rows* and *Missing %* quantify measurements absent within existing files (excluding entirely missing files).

**Table 15:** Completeness of accelerometer data files for each monitored laundry appliance.

ID	Type	Brand	Model	Total expected	Existing	Missing	Missing (%)
1	Wm/Dr	Hoover	WDYN654D-30S	66	48	18	27.27
2	Wm	Samsung	WW80T554DTW	11	11	0	0.00
3	Wm	Electrolux	EWF1272EOW	72	65	7	9.72
4	Wm	Whirlpool	FMG823B	22	22	0	0.00
5	Wm	AEG	L6FBG141	16	16	0	0.00
6	Dr	Beko	DHS7412PA0	18	17	1	5.56
6	Wm	Samsung	WF80F5E0W2W	20	6	14	70.00
7	Wm	Balay	3TS866EE	13	7	6	46.15

The *Total Expected* column indicates the number of accelerometer recordings that should have been collected, one for each main experiment file. *Found* counts the number of accelerometer files actually present and containing valid data, while *Missing* and *Missing %* quantify cases where the entire accelerometer file is absent.

**Table 16:** Within-file completeness of accelerometer recordings for each monitored laundry appliance.

ID	Type	Brand	Model	Expected rows	Actual rows	Missing rows	Missing (%)
1	Wm/Dr	Hoover	WDYN654D-30S	45,449,979	25,047,815	20,402,164	44.89
2	Wm	Samsung	WW80T554DTW	7,920,307	7,799,853	120,454	1.52
3	Wm	Electrolux	EWF1272EOW	104,785,681	104,320,512	465,169	0.44
4	Wm	Whirlpool	FMG823B	35,755,487	33,950,705	1,804,782	5.05
5	Wm	AEG	L6FBG141	40,769,693	8,748,875	32,020,818	78.54
6	Dr	Beko	DHS7412PA0	52,535,599	27,516,030	25,019,569	47.62
6	Wm	Samsung	WF80F5E0W2W	7,239,184	7,131,078	108,106	1.49
7	Wm	Balay	3TS866EE	8,546,990	8,356,795	190,195	2.23

*Expected rows* are calculated from each file’s start-end duration and nominal sampling frequency. *Actual rows* are the number of unique samples present. *Missing rows* and *Missing %* quantify missing samples within existing files (excluding entirely missing files).

#### Implications of missing data

The implications of missing data reported in Tables 12 and 13 are analysed using four use case domains selected as examples: multimodal fault detection, appliance characterisation, soft-sensor development, and environmental sensitivity studies.

- **Multimodal Fault Detection:** While sensor availability varies across the dataset (e.g., audio was recorded exclusively at station M1), a “usable intersection” of data for sensor fusion tasks was verified. Specifically, the dataset contains **246 complete cycles** featuring synchronised energy, vibration, and audio data, creating a multivariate structure essential for fusion fault classification approaches^43^. From this total, a subset of **216 cycles** is particularly valuable for predictive maintenance research^44^, comprising 131 cycles from a healthy machine (Becken BWM5381IX) and 85 cycles from a faulty unit of the same model. This enables robust comparative analysis and model training without data imputation.
- **Appliance Characterisation (NILM):** Considering the creation of ground-truth references for Non-Intrusive Load Monitoring (NILM)^45^, the impact of missing data is minimal. The core electrical signals (Current, Voltage, Power) in the laboratory dataset exhibit only 0.18% missingness, attributed to time drifts of single values that can be ignored or easily interpolated. This ensures high-resolution appliance “fingerprints” that can be used to train and validate disaggregation algorithms without the interference of aggregate household noise or signal gaps.
- **Soft-Sensors:** The development of soft-sensors such as those used for virtual laundry mass estimation^46,47^, moisture content monitoring^48^, or energy performance assessment^28^ requires signal continuity to accurately model physical states. As demonstrated in Table 12, the variables required for this task (Voltage, Current, and Power) exhibit 0% structural loss in the laboratory, meaning all the necessary features are present in every experiment file. Furthermore, Table 13 confirms that signal continuity is preserved, with only 0.18% of individual samples missing.
- **Environmental sensitivity studies:** Analysis of environmental factors is robust due to high sensor reliability and data redundancy. The structural missingness for laboratory environmental sensors (ambient_temperature, ambient_humidity) is minimal (0.93%), and signal continuity remains high with only 0.07% of samples missing. This data enables bidirectional environmental research: quantifying thermodynamically relevant correlations, such as the influence of inlet temperature on energy demand^49,50^, and assessing the appliance’s resource footprint on the ecosystem^51^. To support these types of analysis, the water inlet sensor maintains low missingness (0.08% of samples - see Table 13), and it is complemented with external weather data.

In the household dataset, the amount of missing data is higher (12.88%) in the energy and operational data, and 7.87% in the accelerometer data. These missing values are due to the combination of the chosen data acquisition methodology and intermittent failures of the cellular communication network. Even so, in addition to the remaining data that can be used for real-life energy-related use cases, the dataset can serve as a benchmark for validating the robustness of data imputation^52^ and resilient learning^53^ algorithms in the context of the IoT.

### Power grid readings

**Fig. 9:**
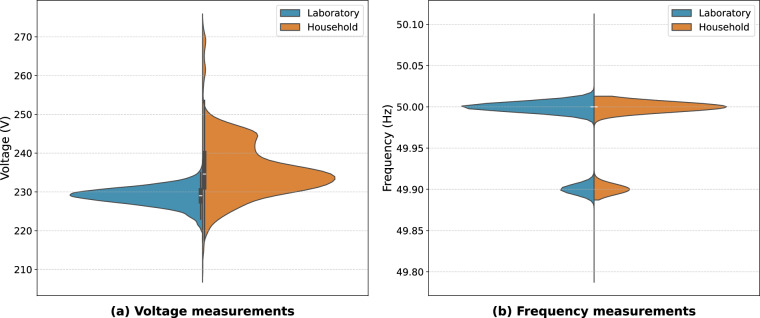
Comparative analysis of (**a**) voltage and (**b**) frequency stability in laboratory and household environments relative to IEC 60038 requirements.

In Portugal, the electricity grid follows the IEC 60038 international standard, providing a standardised frequency of 50 Hz and an end-user service voltage of 230 V with a tolerance of 10%. Thus, the voltage can range from 207 V to 253 V^54^. Figure 9(a) compares laboratory and household dataset voltage readings. They fall within the standard’s expected boundaries, with most values closely clustered around the nominal voltage. Although some outliers suggest occasional fluctuations, all values remain within acceptable tolerance limits. Voltage measurements obtained in the laboratory exhibit greater stability than those recorded in residential settings. This observation is expected, as lower-density dormitory areas outside the city centre are more susceptible to voltage instability due to ageing or outdated electrical grid infrastructure^55^.

As for the frequency, Fig. 9(b) presents a violin plot summarising the distribution of frequency readings of all samples in the laboratory and household environment datasets (the exploratory stage dataset does not contain frequency readings.) Most values are tightly centred around the nominal frequency of 50 Hz, showing minimal variation of 49.90 Hz and a minimal amount of outliers at 50.10 Hz and 49.80 Hz. However, these values remain well within the acceptable tolerance limits. This shows that the readings are consistent with the IEC 60038 standard.

### Washing machine energy and water consumption validation

The energy consumption measurements of this work’s tests are compared with the manufacturer’s published data, which were calculated in accordance with EU Commission regulations. Table 17 presents a side-by-side comparison of the laboratory washing machines’ rated power, eco 40–60 weighted energy consumption (*E*_W_), Energy Efficiency Index (*EEI*), Energy Efficiency Class (*EEC*), and weighted water consumption (*W*_W_).

**Table 17:** Comparison of laboratory washing machine energy consumption: manufacturer specifications vs. laboratory measurements.

Brand	Model	Env.	Rated power (W)		*E*_W_ (Eco 40–60)		*EEI*		*EEC*		*W*_W_ (Eco 40–60)	
			Man.	Obs.	Man.	Obs.	Man.	Obs.	Man.	Obs.	Man.	Obs.
Becken	BWM5379IX	Lab.	2000	1836	0.545	0.345	60	38	B	A	48	40
Becken	BWM5381IX	Lab.	2000	1853	0.628	0.563	60	53	B	B	57	51
Becken-flt	BWM5381IX	Lab.	2000	1853	0.628	0.553	60	53	B	B	57	48
Kubo-1	KBWM8321	Lab.	2000	2008	0.737	0.659	91	82	E	E	43	41
Kubo-2	KBWM8321	Lab.	2000	2032	0.737	0.954	91	118	E	G	43	46
Kunft	KWM5317	Lab.	2000	2117	0.827	0.908	91	99	E	F	48	48
Kunft	KWM5316	Lab.	2000	2007	0.784	0.853	91	98	E	F	45	44
AEG	L6FBG141	Hs.	2200	2135	—	—	—	—	—	—	—	—
Balay	3TS866EE	Hs.	2300	2125	—	—	—	—	—	—	—	—
Electrolux	EWF1272EOW	Hs.	2200	2195	—	—	—	—	—	—	—	—
Hoover	WDYN654D-30S	Hs.	2150	2074	—	—	—	—	—	—	—	—
Hotpoint	FMG823B	Hs.	1850	1883	—	—	—	—	—	—	—	—
Samsung	WW80T554DTW	Hs.	2300	2058	—	—	—	—	—	—	—	—
Samsung	WF80F5E0W2W	Hs.	2400	2107	—	—	—	—	—	—	—	—

**Man**.: Manufacturer; **Obs**.: Observation; ***EEI***: Energy Efficiency Index; ***EEC***: Energy Efficiency Class; ***W***_**W**_: Weighted water consumption; **Env**.: Environment (Lab vs. Household).

For the rated power, the observed values were obtained by averaging the peak power consumption of all the water-heated test runs. For household washing machines, only the rated power values can be compared with manufacturer specifications, as information on cycle class, heating settings, and laundry load is not available. *E*_W_, *EEI*, and *W*_W_ were calculated by using eco-design energy label calculations specified in the EU Commission Regulation 2019/2023^56^ and shown in Equations 7, 8, and 10. For *EEI*, the weighted energy consumption of the *eco 40–60* programme at the rated washing capacity, half the rated washing capacity, and a quarter of the rated washing capacity is compared to its standard cycle energy consumption:

7 $$EEI=(\frac{{E}_{{\rm{w}}}}{SC{E}_{{\rm{w}}}})\times 100$$

*E*_W_ is the weighted energy consumption of the washing cycle. *SCE*_W_ is the standard cycle energy consumption in kWh per cycle, rounded to three decimal places. It is calculated as,

$$SC{E}_{{\rm{w}}}=-0,0025\times {c}^{2}+0,0846\times c+0,3920,$$

where c is the washing machine’s rated capacity for the eco 40-60 programme. *E*_W_ is calculated in kWh per cycle, rounded to three decimal places:

8 $${E}_{{\rm{w}}}=A\times {E}_{{\rm{w}},{\rm{full}}}+B\times {E}_{{\rm{w}},1/2}+C\times {E}_{{\rm{w}},1/4}$$

*E*_w,full_, *E*_w,1/2_, *E*_w,1/4_ represent the energy consumption of a washing machine using the *eco 40–60* program at the rated capacity, half capacity, and quarter capacity, respectively. *A*, *B*, and *C* are the weighting factors for the rated (full), half, and quarter washing capacities. The values of the weighting factors depend on the rated capacity according to the following equations:

9 $$\begin{array}{rcl}A & = & -0,0391\times c+0,6918\\ B & = & -0,0109\times c+0,3582\\ C & = & 1\,-\,(A+B)\end{array}$$

The weighted water consumption *W*_W_ of a washing machine is calculated in litres and rounded to the nearest integer as follows:

10 $${W}_{{\rm{W}}}=A\times {W}_{{\rm{w}},{\rm{full}}}+B\times {W}_{{\rm{w}},1/2}+C\times {W}_{{\rm{w}},1/4}$$

*W*_w,full_, *W*_w,1/2_, and *W*_w,1/4_ represent the water consumption (in litres, rounded to one decimal place) of a washing machine using the *eco 40–60* program at rated capacity, half capacity, and quarter capacity, respectively. *A*, *B* and *C* are the weighting factors, described in Equation 9.

Table 18 presents the *EEI* ranges and corresponding energy efficiency classes used to label washing machines accordingly to the EU regulations^56^.

**Table 18:** Washing machine energy efficiency classification based on *EEI* ranges.

*EEI* Range	*EEI* ≤ 52	52 < *EEI* ≤ 60	60 < *EEI* ≤ 69	69 < *EEI* ≤ 80	80 < *EEI* ≤ 91	91 < *EEI* ≤ 102	*EEI* > 102
**Class**	A	B	C	D	E	F	G

The calculation of *EEI* requires full-load observations. However, the dataset lacks observations at the maximum rated capacity. Therefore, the observations at the rated capacity minus 1 kg were used. Regulations specify ambient air conditions of 20 ±2 °C and 65 ±5% relative humidity but the dataset contains cotton and eco program samples measured at 16 ±2 °C (cold) and 31 ±2 °C (hot). To address this, *W*_w,full_, *W*_w,1/2_, and *W*_w,1/4_ values from both temperature conditions were averaged before being applied in Equation 8. The same approach was used for calculating the weighted water consumption.

The majority of observations in Table 17 differ from those declared by the manufacturers by less than 10%, which is allowed per Commission Regulation (EU) 2019/2023 (2019)^56^. However, a more significant reduction was observed in the water and energy consumption of Becken washing machines compared to the manufacturer’s specifications. One potential explanation for the observed 14.3% reduction in water usage is the difference in laundry load during testing. In the dataset measurements, the rated capacity minus 1 kg might not have activated the full-load washing, thereby reducing water intake. Consequently, the lower water consumption likely led to decreased energy usage, as water heating represents the most energy-intensive phase of the washing cycle.

Another noteworthy observation is that the Kubo washing machines were identical in design and manufacturer specifications. However, Kubo-2 exhibited not only significantly higher electricity consumption, but also increased water usage compared to Kubo-1 and the manufacturer’s declared values. Since water heating accounts for the majority of energy use in a washing cycle, the higher water consumption may have contributed to the elevated energy consumption. A malfunctioning heating element or control system could be a contributing factor, although this has not been confirmed. This discrepancy led to a two-class drop in the observed energy efficiency rating.

### Exploratory stage washing machines

To validate the exploratory stage dataset records, observed performance metrics of various washing machine models were compared against values stated in the respective manufacturers’ booklets, as shown in Table 19. Observations were recorded during the water heating phase to calculate the maximum power as the average of the instantaneous peak power readings. Energy and water consumption values were closely aligned with the manufacturer-stated load capacities. However, exact replication was limited by the absence of detailed load data. Several discrepancies emerged due to inherent limitations. Firstly, manufacturers’ lack of a standardised reporting framework led to ambiguity regarding test conditions, such as ambient temperature, water hardness, inlet water temperature, and clothing typology. Secondly, rated power figures were affected by variations in electrical supply, as washing machines typically operate within a 220–240 V range, influencing peak power behaviour. Thirdly, critical information such as manufacturer-declared energy or water consumption was not disclosed in some cases, preventing direct comparison. Despite these limitations, the dataset offers a representative empirical basis for characterising machine performance under realistic operating conditions.

**Table 19:** Observed versus manufacturer-provided values for washing machine performance during the exploratory stage.

Washing machine	Program	Load (kg)		Rated power (W)		Enegy cons. (kWh)		Water cons. (L)	
		Man.	Obs.	Man.	Obs.	Man.	Obs.	Man.	Obs.
Bosch WFO2063EE	Cotton 40	6.00	5.70	2300	2367	0.65	0.47	60.0	65.6
Bosch WFO2063EE	Cotton 60	5.00	3.91	—	—	0.95	0.83	60.0	53.4
Bosch WFO2063EE	Cotton 90	6.00	4.00	—	—	2.10	1.81	67.0	61.3
Indesit IWC71252	Cotton 40	7.00	5.74	1850	2094	0.81	0.53	76.0	49.0
Samsung WF1802WPC	Cotton 40	4.00	3.93	2000-2400	2246	0.57	0.43	Unknown	53.0
Samsung WF1802WPC	Cotton 60	4.00	3.93	—	—	0.77	0.95	Unknown	54.0
Siemens WM12N278EP	Cotton 40	Unknown	7.54	2300	2140	—	0.84	Unknown	72.0
Siemens WM12N278EP	Cotton 40	Unknown	3.94	—	—	—	0.78	Unknown	69.0
Siemens WM12N278EP	Cotton 60	Unknown	3.90	—	—	—	1.15	Unknown	67.0
LG F4WV710P1	Cotton 40	5.25	5.75	Unknown	2224	0.56	0.49	41.0	76.0
LG F4WV710P1	Cotton 60	5.25	3.96	—	—	0.63	1.39	41.0	68.0

**Man**.: Manufacturer; **Obs**.: Observation.

### Tumble dryer energy consumption validation

Following the validation approach used for washing machines, the observed data for dryers were compared with the manufacturer’s specifications. Table 20 compares idle power, rated power, power consumption for the cotton program under half and full-load conditions, energy efficiency index, and energy efficiency class. The manufacturer’s values were obtained from the booklet supplied with the appliance.

**Table 20:** Comparison between the manufacturer’s declared dryer machines’ energy consumption and the laboratory observations.

Brand	Model	Env.	Idle power (W)		Power (W)		Ctn-FL (kWh)		Ctn-HL (kWh)		*EEI*		*EEC*	
			Man.	Obs.	Man.	Obs.	Man.	Obs.	Man.	Obs.	Man.	Obs.	Man.	Obs.
Becken	BDM8331	Lab.	0.8	0.9	800	820	1.90	1.5	1.10	0.90	—	25	A++	A++
Becken	BDM2741	Lab.	0.8	1.4	850	760	1.98	1.7	1.08	0.96	—	27	A++	A++
Beko	DHS7412PA0	Hs.	—	—	850	782	—	—	—	—	—	—	—	—

**Env**.: Environment; **Man**.: Manufacturer; **Obs**.: Observation; ***EEI***: Energy Efficiency Index; ***EEC***: Energy Efficiency Class; **Ctn-FL**: Cotton full load; **Ctn-HL**: Cotton half load.

The rated power was obtained by averaging peak power values observed during the standard cotton program. The energy consumption (kWh) of the cotton drying programs was calculated by averaging the energy consumption observations for half-load and full-load cycles, using experimental samples from both hot and cold environments. The rated power measurements are within the range $[-10.06 \% ,\,2.5 \% ]$ of the manufacturer’s reference.

The methodology for calculating rated power, the variability of the grid voltage, and ambient factors could possibly explain the deviations obtained.

The *EEI* was calculated, and the corresponding efficiency class was assigned according to Delegated Regulation (EU) 392/2012^57^, as this standard was originally used for evaluating the tumble dryers. To calculate the *EEI* of a tumble dryer model, the weighted annual energy consumption for the standard cotton program at full and partial load is compared to the standard annual energy consumption,

$$EEI=\frac{A{E}_{{\rm{C}}}}{SA{E}_{{\rm{C}}}}\times 100,$$

where *AE*_C_ refers to the weighted annual energy consumption and *SAE*_C_ represents the standard annual energy consumption.

(*AE*_C_) is calculated in kWh/year (rounded to two decimal places) by the equation,

$$A{E}_{{\rm{C}}}={E}_{{\rm{t}}}\times 160+\frac{\{({P}_{1}\times {T}_{1}\times 160)+{P}_{0}\times [525600-({T}_{{\rm{t}}}\times 160)-({T}_{1}\times 160)]\}}{60\times 1000},$$

where *T*_1_ is the duration of the ‘left-on mode’ for the standard cotton program at full load, measured in minutes and rounded to the nearest whole minute. *T*_t_ weighted programme time and is rounded to the nearest minute, calculated by the equation,

$${T}_{{\rm{t}}}=\frac{(3\times {T}_{{\rm{dry}}}+4\times {T}_{{\rm{dry}}}^{1/2})}{7},$$

where *T*_dry_ is the programme duration for the standard cotton programme at full load, in minutes (rounded to the nearest minute) and ${T}_{{\rm{dry}}}^{1/2}$ is the programme duration for the standard cotton programme at partial load. The weighted energy consumption (*E*_t_) is calculated in kWh (rounded to two decimal places) by,

$${E}_{{\rm{t}}}=\frac{(3\times {E}_{{\rm{dry}}}+4\times {E}_{{\rm{dry}}}^{1/2})}{7},$$

where *E*_dry_ represents the energy consumption of the standard cotton programme at full load, while ${E}_{{\rm{dry}}}^{1/2}$ corresponds to the energy consumption at partial load. Both values were obtained by averaging observations from both hot and cold environments and rounded to two decimal places. The same approach was applied to drying time.

*SAE*_C_ is calculated as,

$$SA{E}_{{\rm{C}}}=140\times {c}^{0.8},$$

where *c* is the rated capacity of the tumble dryer for the standard cotton programme.

The energy efficiency class of a tumble dryer is determined based on its *EEI*, as specified in Table 21. Although the exact *EEI* value is unavailable, the conversion from the calculated *EEI* to the corresponding energy efficiency class aligns with the classification provided by the manufacturer.

**Table 21:** Transposed dryer energy efficiency classes.

*EEI* Range	*EEI* < 24	24 ≤ *EEI* < 32	32 ≤ *EEI* < 42	42 ≤ *EEI* < 65	65 ≤ *EEI* < 76	76 ≤ *EEI* < 85	85 ≤ *EEI*
**Class**	A+++	A++	A+	A	B	C	D

### Water supply temperature

**Fig. 10:**
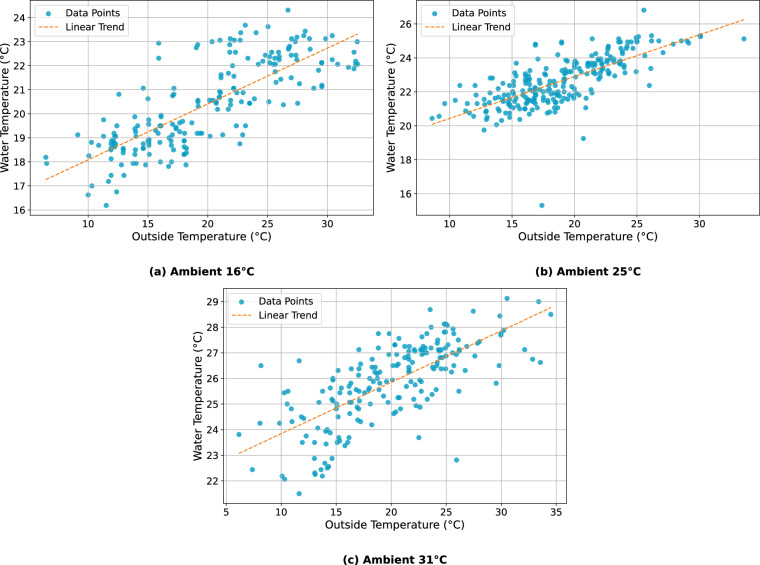
Correlation between outside temperature and water inlet temperature across different laboratory ambient temperature settings.

During the laboratory data acquisition experiments, the inlet water temperature was recorded. The water supplied to buildings is typically stored in large reservoirs, elevated towers, or local storage tanks. As a result, it can be assumed that weather conditions influence water supply temperature. Under this hypothesis, to validate the measurements, a correlation analysis was conducted between outside air temperature data obtained from OpenWeatherMap^58^ and measured water inlet temperature. Figure 10 illustrates the correlation between outside air and water supply temperatures under controlled laboratory ambient temperatures of 16, 25, and 31 °C, respectively.

The maximum inlet water temperature value observed in each washing cycle was used for the analysis at 16 °C, and the minimum was used for the analysis at 25 °C and 31 °C. The Pearson correlation coefficient (PCC)^59^ was 0.784, 0,725, and 0.719, and the coefficient of determination (*R*^2^)^60^ was 0.615, 0.530, and 0.517, for the three ambient temperatures considered. The results indicate a consistent, moderately strong, positive correlation between outdoor air temperature and inlet water supply temperature. In colder environments, water temperature is more sensitive to fluctuations in outside temperature, as indicated by a steeper slope in the trend. Conversely, water temperature remains more stable in hotter ambient conditions, suggesting that external heating effects are less pronounced.

### Water drain temperature

**Fig. 11:**
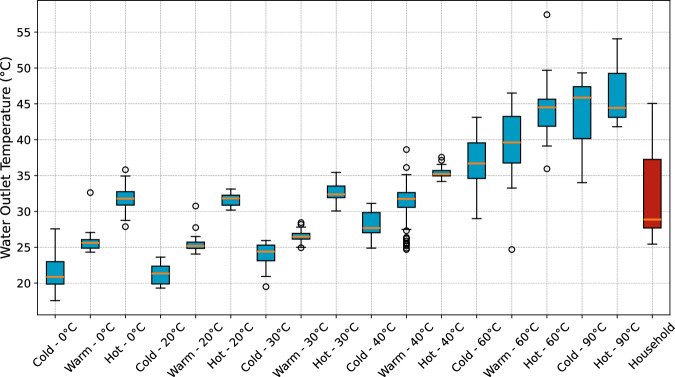
Distribution of each washing cycle maximum recorded drain water temperature over programmed cycle heating temperatures in the laboratory. The laboratory ambient temperature setting is also shown on the horizontal axis. The rightmost box plot illustrates the distribution obtained in the household washing cycles for which the heating temperature is unknown.

The water drain temperatures measured in the laboratory were validated by comparing them with the washing machine’s heating temperature during the washing cycles. Figure 11 plots the distribution of the maximum recorded water outlet temperatures of each washing cycle (water_temperature_out), broken down by increasing cycle heating temperature set-points (program_heat in aggregated_data.csv). The figure considers only washing cycles with deterministic water heating patterns. The Eco 40–60 program is excluded, as its heating behaviour and final temperature vary significantly depending on the appliance brand and the laundry load, making it unsuitable for consistent comparison.

The data confirm that cycles with water heating set to 0 °C and 20 °C do not exhibit temperatures beyond what ambient room temperature and water supply temperature might impose. The influence of ambient room temperature is noticeable, particularly at lower heating set points. For cycles with higher heating levels (30 °C to 90 °C), as expected, the measurements successfully detect elevated outlet temperatures. In household observations, the maximum outlet temperatures of each cycle range from approximately 25 °C to 45 °C. When compared with those obtained under controlled laboratory conditions, it can be inferred that the corresponding washing programs operate within a nominal range of 40 C to 60 °C, which is consistent with common household washing practice and with the Eco 40–60 options.

### Washing machine drum door temperature

**Fig. 12:**
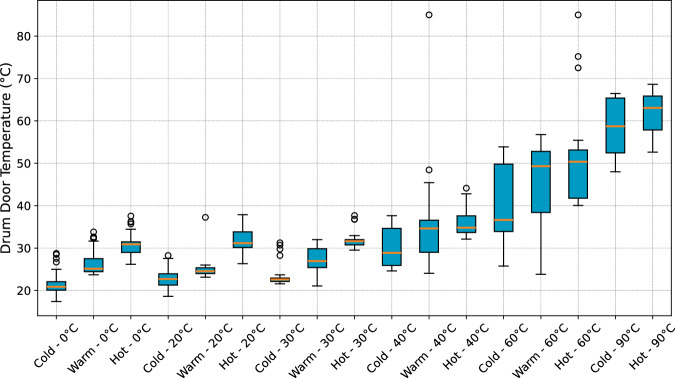
Distribution of each washing cycle maximum drum door temperature over cycle heating temperatures programmed in the laboratory. The laboratory ambient temperature setting is also shown on the horizontal axis.

The drum door temperatures measured in the laboratory were validated by comparing them with the washing machine’s cycle heating temperature set-point. Figure 12 plots the distribution of the maximum drum door temperature of each washing cycle (drum_door_temp), broken down by increasing cycle heating temperature set-points (program_heat in aggregated_data.csv). In comparison to water outlet hose temperature readings, as expected, the drum door temperatures are closer to the heating set-point temperature of the selected program. As for the hose temperature readings, the ambient temperature setting influences the temperature readings. These data confirm that cycles set to 0 °C and 20 °C do not exhibit additional heating, aligning with ambient room temperature.

### Washing machine drum temperature

**Fig. 13:**
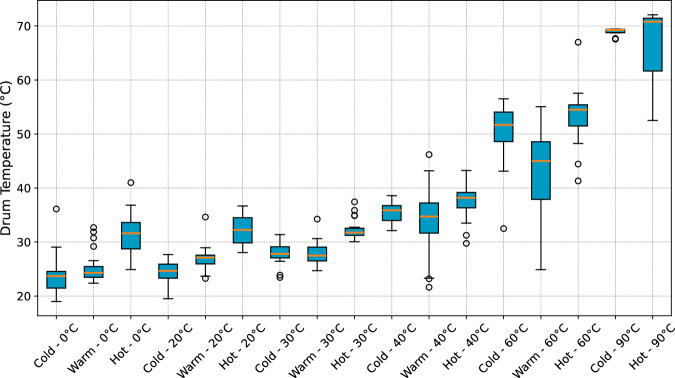
Distribution of each washing cycle maximum drum temperature over cycle heating temperatures programmed in the laboratory. The laboratory ambient temperature setting is also shown on the horizontal axis.

The drum temperatures measured in the laboratory were validated by comparing them with the washing machine’s cycle heating temperature set-point. Figure 13 plots the distribution of the maximum drum temperature of each washing cycle (drum_temperature), broken down by increasing cycle heating temperature set-points (program_heat in aggregated_data.csv).

The data confirms that cycles set to 0 °C and 20 °C do not exhibit additional active heating, remaining influenced by ambient room temperature and inlet water temperature. The general behaviour presented in Fig. 12 is also visible, although possibly with more interference of ambient temperature and heat generated by the motor during operation, which is consistent with the sensor’s placement in the outer shell of the drum near the motor, where heat accumulation can occur.

### Lint trap temperature

Two different temperature sensors were placed inside the lint trap chamber to observe the temperature inside the dryer. The manufacturer-provided booklets do not disclose target temperatures for specific cycles or general operating heat. Generally, tumble dryers operate at drying temperatures between 35 °C and 60 °C, depending on the specific design and drying conditions^61^.

**Fig. 14:**
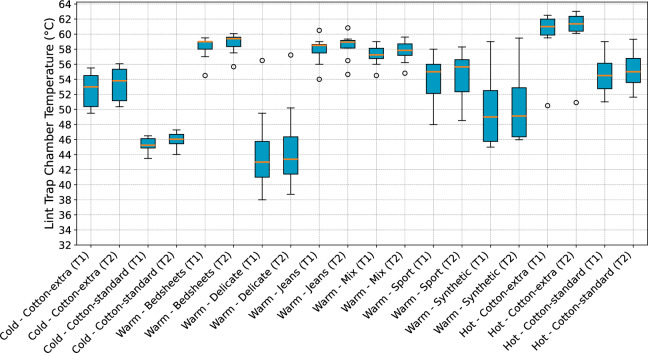
Distribution of each drying cycle maximum lint trap temperatures of the Becken BDM8331 dryer, over cycle programmed in the laboratory and ambient temperature. For the same programme and ambient temperature, the boxplots of the two sensors are shown side by side.

**Fig. 15:**
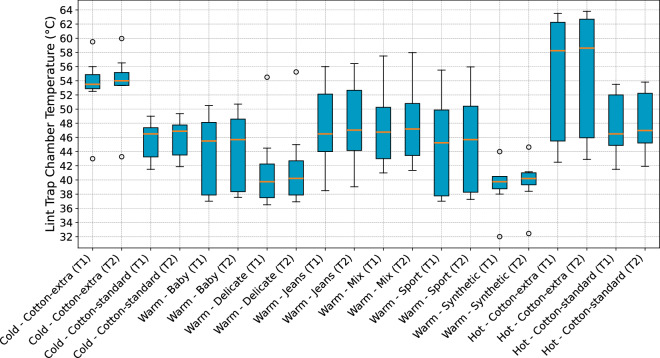
Distribution of each drying cycle maximum lint trap temperatures of the Becken BDM2741 dryer, over cycle programmed in the laboratory and ambient temperature. For the same programme and ambient temperature, the boxplots of the two sensors are shown side by side.

Based on the observations in Figs. 14 and 15, each for one of the two available dryers, it is evident that ambient temperature extremes, such as “hot” (ambient temperature set to 31 °C) and “cold” (ambient temperature set to 16 °C), influence the maximum temperatures reached inside the drying machines. This effect is particularly noticeable in the BDM2741 machine’s *Cotton-Extra* program, exhibiting the widest temperature range, spanning from 42.5 °C to 63.5 °C. Both figures show that the measurements of the two sensors are highly consistent across ambient temperature and programme settings, with only a slight offset between the box plots.

**Fig. 16:**
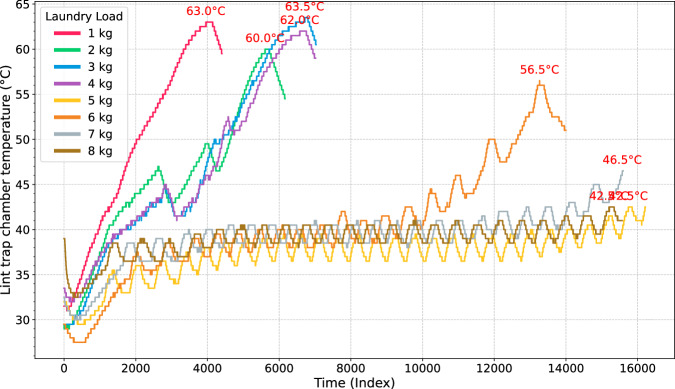
Lint trap chamber temperature during the *Cotton-Extra* drying program for different laundry loads in the Becken BDM2741 dryer.

Figure 16 illustrates examples of the maximum temperatures reached during the Becken BDM2741 *Cotton-Extra* drying cycle for different laundry loads.

**Fig. 17:**
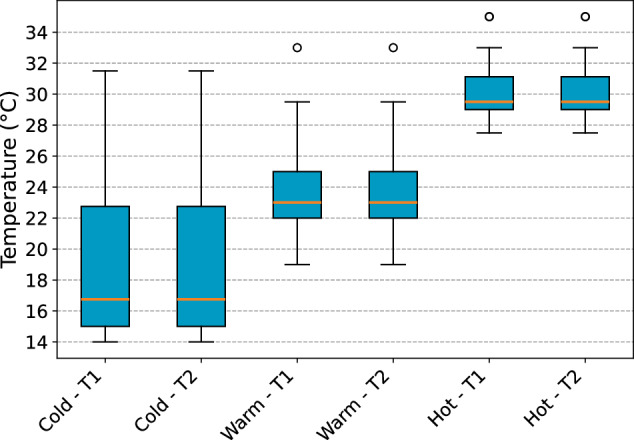
Comparison of the distributions of initial lint trap temperatures of two sensors across different ambient temperature settings.

In general, lighter loads reach higher peak temperatures (60–63.5 °C) and complete their cycles sooner, whereas heavier loads operate at lower, more fluctuating temperatures (42.5–56.5 °C) and complete the cycles later. The data also confirms the consistency of the temperature readings: at the beginning of the cycle, most loads start between 30–33 °C, except for the 8 kg load, which initially registers at 40 °C. To further validate the temperature measurements, the initial temperature of the lint trap chamber is expected to closely approximate the ambient temperature, thereby serving as a baseline or ground truth. Figure 17 presents the initial temperature measurements for all drying cycles conducted under varying ambient conditions using two distinct temperature sensor models.

On average, the difference between the T1 and T2 sensors placed within the lint trap chamber remained between 0.4 °C and 1 °C. Based on the observations, the initial temperatures clearly show the influence of the ambient setting. Although the median temperature was within the acceptable range across all ambient settings, the data showed a broader spread in the “cold” environment compared to the distributions observed under “hot” and “warm” conditions.

### Ambient Temperature

**Fig. 18:**
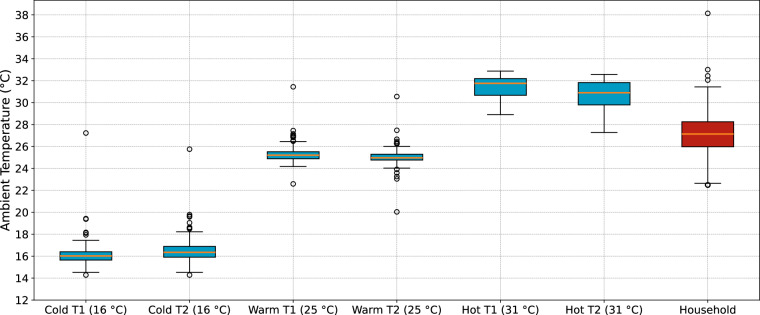
Boxplots showing the distributions of ambient temperature readings collected by sensors T1 and T2 during the execution of washing machine cycles, under controlled laboratory conditions. T1 and T2 were placed at different locations. The rightmost box plot displays the first temperature observation from each washing cycle collected in households.

Figure 18 presents the distributions of ambient temperature readings made during the washing machine cycles conducted under the three air-conditioned laboratory setpoints: 16 °C (cold), 25 °C (warm), and 31 °C (hot).

The measurements reflect the environmental control conditions under which the data were collected. The boxplots indicate that the laboratory closely maintained the target temperatures, with only minor deviations and a few outliers, mostly associated with temperature drifts when switching between setpoints (e.g., from cold to warm) and the influence of outside weather temperatures. The temperature readings of the two sensors differed by less than 0.5 °C on average. This slight deviation suggests a uniformly controlled ambient environment. Notably, T1 was positioned opposite the air conditioning unit, while T2 was located near the centre of the room, indicating the system’s effectiveness in maintaining spatial temperature consistency.

To illustrate the household environment ambient temperature, the first measurement of each cycle was selected as the best representative of the actual ambient conditions. The household acquisition kit and its temperature sensor were positioned at the back of the appliance, where heat tends to accumulate due to the appliance being enclosed by furniture or a special building structure. During operation, such conditions would likely increase temperature, compromising the correlation with the actual ambient temperature. Using only the initial measurement of each cycle minimises this unwanted influence. Observed temperatures ranged from 23 °C to 31 °C, which is within the expected normal range for the country’s warm/hot climate.

### Pressure

**Fig. 19:**
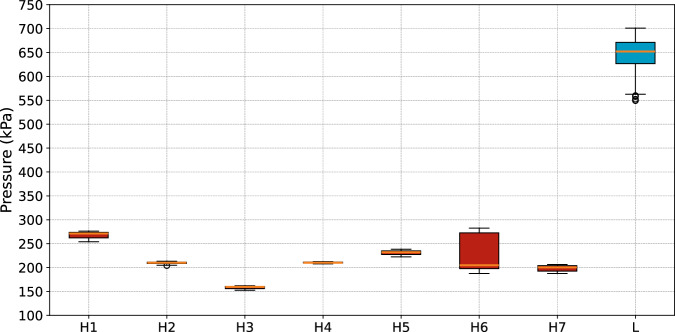
Distribution of water pressure measurements across laboratory (L) and household environments (H1-H7, as defined in Table 3).

Water pressure monitoring was conducted in the laboratory and residential environments. Typical residential water pressure ranges from 200 to 400 kilopascals (kPa), with 100 kPa considered the minimum acceptable threshold for functionality and comfort in domestic water plumbing systems^62^. As illustrated in Fig. 19, the majority of pressure readings from the household deployments (H1-H7) fall within the expected range, with measurements from 200 to 300 kPa, indicating consistent and realistic data acquisition. Minor household variability is observed, reflecting typical fluctuations in municipal supply systems and household demand patterns. In contrast, the laboratory (L) exhibits a significantly higher pressure, centred around 650 kPa. This higher value is consistent with expectations for institutional or commercial plumbing infrastructure, which often requires greater water delivery capacity and flow rates to support simultaneous or intensive usage scenarios. The box plot representing the laboratory data shows a wider interquartile range and a greater overall spread than the household measurements, which can be a consequence of the significantly larger volume of observations collected over a longer monitoring period in the laboratory setting.

### Vibrations

Washing machines have varying spinning speeds depending on the rated rotations per minute (rpm) and the selected program. The rated rotation speed for each experiment is noted within the aggregated_data_acc.csv meta file, ranging from 600 to 1400 rpm. Accelerometer data were validated using washing samples with a 0 kg laundry load, serving as a baseline for identifying dominant vibration frequencies and harmonics during the centrifugation phase. The expected dominant frequencies were calculated based on the drum rotation speed, ranging from approximately 10.00 Hz at 600 rpm to 23.33 Hz at 1400 rpm. To extract the dominant frequency components, a Fourier transform was applied to the accelerometer signals captured during the final centrifuge stage, when the drum reached its maximum rotation speed, as defined by the selected washing program. This methodology was applied to a selection of washing machines with documented rotation speeds for specific programmes.

Table 22 summarises the expected and detected dominant frequencies along each accelerometer axis during the centrifugation phase. Despite axis-dependent sensitivity, most accelerometer axes consistently produced dominant frequency components or harmonics aligned with the expected dominant frequencies derived from the drum’s rotational speed, supporting the validity of the collected data. In most cases, the fundamental frequency and its harmonics up to the fourth order were observed as the most prominent components. The accelerometer mounted on the back of the machine has its Z-axis oriented perpendicular to the rear panel, pointing directly toward the centre of the drum. As a result, the Z-axis aligns with the drum’s axis of rotation during centrifugation, making it less sensitive to vibrations at the expected dominant frequency, which is confirmed by the results in the **BZ** column of Table 22. Additionally, the 50 and 100 Hz frequencies were occasionally detected, likely corresponding to the electrical grid frequency and its first harmonic, although they could also be related to the expected frequencies of 10.0 or 23.33 Hz.

**Table 22:** Expected and detected dominant frequencies during the centrifuge (spin) cycle of a selection of washing machines and programmes.

Washer	Rpm	Exp. Hz	Program	TX	TY	TZ	BX	BY	BZ	SX	SY	SZ
BWM5379IX	600.0	10.0	Wool	**10.40**	**10.40**	**10.40**	52.10	**10.40**	**31.25**	**10.40**	**10.40**	94.24
BWM5379IX	600.0	10.0	Delicate	**10.40**	**10.40**	**10.40**	**10.40**	**10.40**	**31.20**	**10.40**	**10.40**	**31.20**
BWM5381IX	600.0	10.0	Wool	**10.06**	**10.06**	50.00	**10.06**	**10.06**	50.00	**10.06**	**10.06**	**10.06**
BWM5381IX	600.0	10.0	Delicate	50.15	**10.01**	50.15	99.76	**10.01**	99.66	93.75	**10.01**	**30.08**
KWM5317	600.0	10.0	Wool	**30.08**	**10.06**	50.15	**10.06**	**10.06**	7.47	**10.06**	4.20	4.20
KWM5316	600.0	10.0	Wool	**10.16**	**10.16**	5.18	99.17	**10.16**	99.17	98.44	**10.16**	99.61
BWM5379IX	800.0	13.3	Mix	**27.83**	**13.92**	8.69	**13.92**	**13.92**	**27.83**	**13.92**	**27.83**	**13.92**
BWM5379IX	800.0	13.3	Sport	**27.88**	**13.92**	**41.80**	**13.92**	**13.92**	**41.80**	**13.92**	**27.88**	**41.8**
BWM5381IX	800.0	13.3	Mix	**13.33**	**13.33**	49.90	**13.33**	**13.33**	**26.66**	**13.33**	100.00	**26.66**
BWM5381IX	800.0	13.3	Sport	**13.33**	**13.33**	49.95	**13.33**	**13.33**	49.95	0.05	99.71	**13.33**
KWM5317	800.0	13.3	Cotton 20	51.90	**13.72**	7.81	**13.77**	2.64	51.9	**13.77**	90.28	3.03
KWM5316	800.0	13.3	Cotton 20	**13.13**	98.39	98.39	**13.13**	47.27	47.27	**13.13**	45.95	98.05
BWM5379IX	1000.0	16.6	20	**16.70**	**16.70**	**16.70**	82.37	**16.70**	50.10	**16.70**	**16.7**	**16.70**
BWM5381IX	1000.0	16.6	20	**15.97**	**15.97**	**15.97**	**15.97**	**15.97**	47.95	**15.97**	**15.97**	**31.98**
KWM5317	1000.0	16.6	Jeans	6.74	**15.87**	23.24	12.06	8.94	**47.66**	0.88	0.88	5.03
KWM5316	1000.0	16.6	Jeans	**15.97**	**15.97**	99.56	**15.97**	**16.21**	**15.97**	**15.97**	**15.97**	14.55
BWM5379IX	1200.0	20.0	Synthetic	27.78	**20.80**	34.72	34.72	**20.85**	34.72	**20.85**	34.72	34.72
BWM5381IX	1200.0	20.0	Synthetic	**18.70**	**18.70**	**37.40**	**18.70**	**18.70**	**18.70**	**18.70**	**18.70**	**37.40**
BWM5379IX	1400.0	23.3	Eco 40–60	**24.76**	**24.76**	**49.51**	**49.51**	**24.76**	**49.51**	**24.76**	**24.76**	**24.76**
BWM5381IX	1400.0	23.3	Eco 40–60	**24.90**	**24.90**	**48.49**	**24.90**	**24.90**	97.85	**24.90**	53.42	**24.90**

Bold values mark the entry nearest to the expected frequency or its harmonics. Experiments were conducted with an empty drum.

**WM**: Washing Machine; **Exp. Hz**: Expected Frequency; **TX**: Top X-axis; **TY**: Top Y-axis; **TZ**: Top Z-axis; **BX**: Back X-axis; **BY**: Back Y-axis; **BZ**: Back Z-axis; **SX**: Side X-axis; **SY**: Side Y-axis; **SZ**: Side Z-axis.

Supplementary Table 2 presents the full results, including the top three dominant frequencies (f1-f3), accelerometer locations (top, back, side panels), spin speeds (rpm), and the time intervals used for frequency extraction using the Fourier transform.

### Sound recording via pick-up

The audio samples recorded using the pickup sensor were validated using the same approach as for the accelerometer data. Only recordings with a 0 kg laundry load were initially chosen for dominant frequency extraction to reduce variability. However, for a machine KWM5316, the 0 kg configuration resulted in dominant frequencies being obscured by broadband noise concentrated around 100 Hz. A 4 kg load was used for these specific machines to mitigate this issue, improving the signal-to-noise ratio in the relevant frequency bands.

Table 23 presents the expected and detected dominant frequencies and harmonics, based on the spinning speed specified for each specific machine and programme combination, the beginning and end of the time window used for Fourier analysis, and the top ten frequency peaks extracted from the Fourier transform of each recording. Columns f6 and f9 were omitted since they did not contain expected dominant or harmonic frequencies.

**Table 23:** Top ten dominant frequency components extracted from the Fourier transform of washing machine sound recordings made during the centrifugation stage.

Washer	Program	Kg	Rpm	Exp. Hz	Start	End	f1	f2	f3	f4	f5	f7	f8	f10
BWM5381IX	Wool 40	0	600	10.0	3996	3999	**10.09**	1487.14	424.61	1168.18	155.44	987.16	96.23	49.80
BWM5381IX	Delicate 30	0	600	10.0	2644	2654	**10.09**	106.32	425.28	50.47	289.35	96.23	1016.10	1168.85
KWM5316	Wool	4	600	10.0	3754	3758	99.59	**30.28**	499.30	199.86	299.45	69.98	189.76	90.17
BWM5381IX	Mix 40	0	800	13.3	3628	3630	141.31	438.07	158.13	39.70	957.55	**13.46**	483.82	26.92
BWM5381IX	Sport 40	0	800	13.3	2521	2524	141.31	**39.70**	**13.46**	158.13	286.66	938.04	967.65	958.23
BWM5381IX	Intensive 40	0	800	13.3	12903	12907	141.31	438.07	285.99	**13.46**	966.98	1556.45	937.37	158.13
BWM5381IX	Fast 45	0	800	13.3	2602	2606	141.31	40.37	**13.46**	82.77	286.66	438.07	938.04	84.11
KWM5316	Cotton 0	4	800	13.3	7652	7658	39.70	66.62	26.92	100.26	**13.46**	146.69	119.78	106.32
KBWM8321	Cotton 30	0	800	13.3	9048	9053	26.24	702.52	234.17	**39.70**	602.26	718.67	79.40	**13.46**
BWM5381IX	20	0	1000	16.7	3239	3249	**16.15**	1002.64	**32.30**	47.78	169.57	525.54	1050.42	485.17
KWM5316	Mix 40	4	1000	16.7	4926	4936	28.26	100.94	104.97	**15.48**	50.47	102.96	80.75	37.68
KWM5316	Jeans	4	1000	16.7	5664	5668	144.00	111.70	175.63	47.78	495.26	463.64	**16.15**	63.93
KWM5316	Volumous	4	1000	16.7	6001	6011	183.71	499.97	100.26	300.12	**16.15**	1310.83	**32.30**	563.23
BWM5381IX	Synthetic 40	0	1200	20.0	9645	9647	37.68	197.84	**18.84**	612.35	130.55	427.97	55.85	40.37
BWM5381IX	Cotton 30	0	1400	23.3	10444	10450	**44.41**	38.36	99.59	88.15	**22.21**	945.44	132.56	233.50
KWM5316	Cotton 40	0	1400	23.3	10439	10443	**44.41**	38.36	100.26	88.15	**22.21**	132.56	233.50	154.10
KWM5316	Cotton 60	0	1400	23.3	11346	11350	**43.07**	85.46	37.01	**21.53**	150.06	227.44	45.76	171.59
KWM5316	Eco 40-60	0	1400	23.3	10309	10313	**47.10**	**23.55**	251.00	71.33	41.05	981.78	166.21	88.82

Bold values indicate frequencies near the expected dominant frequency or corresponding harmonics. The *Start* and *End* columns delimit the time window used for Fourier analysis.

The expected dominant frequency or the first harmonic emerged as the strongest spectral component in several cases. Higher-order harmonics exhibited greater power in other instances, possibly due to structural resonances. Nevertheless, across all analysed samples, the expected fundamental frequency or one of its harmonics consistently appeared among the top 10 dominant frequency peaks. Only one sample did not produce the dominant frequency within the 10 strongest spectral components. These results confirm the reliability of the recorded audio data and support the validity of the frequency extraction process in capturing the mechanical behaviour associated with drum rotation.

## Usage Notes

The data is contained within three data formats: the primary aggregated sensor data (energy, operational, and environmental) is stored in CSV file format, which can be read by most scientific tools. Accelerometer data are stored in the Apache Parquet format^41^ to efficiently store and retrieve large-scale, columnar time-series data. Parquet is widely supported across many platforms and programming languages. The audio recordings associated with the dataset are stored in lossless FLAC^42^ format to preserve the full fidelity of the original signals.

The Python pandas library is recommended for loading the data, as it provides built-in support for both CSV and Parquet file formats. This enables users to efficiently load and manipulate the dataset using a consistent and widely adopted data analysis framework.

The aggregated_data.csv, aggregated_data_acc.csv and aggregated_data_audio.csv files should be used to filter, select, and access specific data records based on the metadata stored, including file names and paths. Example code snippets are provided in the quick_start.ipynb Jupyter notebook, available in the LARCO dataset GitHub repository. This notebook is intended to help users quickly begin reading and working with the data records.

## Supplementary information

Supplementary_Table_1

Supplementary_Table_2

## Data Availability

The datasets generated and analysed during the current study are available in the Zenodo repository. The dataset is made publicly available via the following persistent identifier 10.5281/zenodo.19666168. Additionally, supplementary weather data retrieved to account for environmental conditions during the laundry processes were sourced from the OpenWeather API (https://api.openweathermap.org).

## Code availability

All the supplementary code files used in this study are available in the LARCO GitHub repository (https://github.com/larco-dataset/utils). It ensures the complete transparency and reproducibility of the data processing and analysis steps in this paper.

The repository includes scripts for:

• reading data files (read_main_data.ipynb, read_acc_data.ipynb, read_audio_data.ipynb),

• labelling experimental phases (auto_water_heating_label.ipynb, manual_centrifugation_label.ipynb),

• identifying missing data (count_missing_data.ipynb), and

• extracting dominant frequencies from both audio and accelerometer files (extract_frequencies_from_audio.ipynb, extract_frequencies_from_accelerometer.py),

• retrieving weather data (weather_data.ipynb)

Additionally, it includes tools for:

• listening to audio files (listen_audio_file.py),

• data visualisation (plotting_data.ipynb),

• computing appliance power usage (calculating_power_usage.ipynb), and

• generating additional statistics and KPIs for the aggregated_data.csv file (compute_extra_statistics.ipynb).

The code relies on a range of standard scientific Python libraries. **NumPy (2.2.6)** and **Pandas (2.2.3)** were used for numerical and tabular data manipulation. **Matplotlib (3.10.3)**, **Plotly (6.1.0)**, and **Librosa (0.11.0)** enabled the creation of static and interactive visualisations. **Soundfile (0.13.1)**, **Sounddevice (0.5.2)**, **Librosa (0.11.0)**, and **SciPy (1.14.1)** provided functionality for reading, playing, and analysing audio data. For building interactive dashboards, the **Dash framework (3.0.4)** was utilized, along with **dash_table (6.0.2)** and **dash_daq (0.6.0)**.
