# Supplementary material for: Household Laundry Appliance Resource Consumption and Operation Dataset
Source: Sci Data. 2026 May 16;13:1131. doi: 10.1038/s41597-026-07361-6 (PMC13439325; doi:10.1038/s41597-026-07361-6)
Supplement: Supplementary file 1 [file 41597_2026_7361_MOESM1_ESM.pdf]

Supplementary Table 1. Overview of publicly available energy monitoring datasets with comparison to LARCO (highlighted). Our dataset extends prior work with high-frequency multi-modal sensing (1Hz–11kHz), detailed appliance operational states, and comprehensive environmental/usage metadata for washing machine performance analysis.

Dataset/ Year	Devices	Samp. Type	N° houses	Samp. Freq.	Energy Vars	Envir. Vars	Oper. Vars	Envir.	User op. Vars	Metadata	Main Use Cases
**LARCO** 2025	WM DR	IL	10	Plug: 1Hz Vib.: 200Hz Sound: 11kHz	P,I,V ϕ,f	T_{in},T_{out} RH_{in},RH_{out} P_{water},P_{atm}	$Q_{\text{w-in}}$,$Q_{\text{w-out}}$ a_{vib}, $T_{\text{w-in}}$ T_{drum},T_{door} $T_{\text{w-out}}$,a_{vib} $T_{\text{lint-trap}}$, V_{pickup}	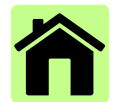 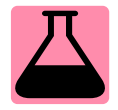	m_{laundry},m_{dry} m_{wet},P_{wash} P_{dry},T_{set}	V_{cond},App_{brand} App_{model},Lbl_{heating} $Lbl_{\text{water-in}}$,$Lbl_{\text{water-out}}$ $Lbl_{\text{centrifuge}}$,m_{wet}	Appliance performance, assessment, optimization, and sustainability
WPuQ Schlemminger et al. 2022	THA HP PV	IL NIL	38	Mains: 10s Plug: 10s	P,Q,S I,U, ϕ	T_{in}, RH_{in} v_{wind},G	HP-compressor HP-heating	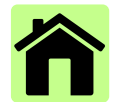	N/A	Occupancy, household attributes, HP performance and capacity,PV integration	HP-integrated load analysis for grid impact
DEDDIAG Wenninger et al. 2021	THA	IL NIL	15	Mains: 1Hz Plug: 1Hz	P,Q,S V,I, ϕ	N/A	N/A	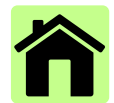	P_{dry} P_{wash}	Annotations - on/off, cycles - start end	NILM, appliance classification, house occupancy
LIFTED Yan et al. 2020	THA	IL	1	Plug: 50Hz	P,I,V Q	N/A	N/A	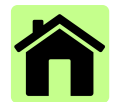	N/A	App$_{\text{brand}}$, App$_{\text{model}}$ annotations - on/off, transient behavior	NILM, appliance level energy disaggregation
BLOND Kriechbaumer et al. 2018	TOA	IL NIL	N/A	Mains: 50-250kSps Plug: 6.4-50kSps	V,I,P S, ϕ, f	N/A	N/A	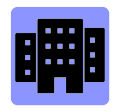	N/A	App$_{\text{brand}}$, App$_{\text{model}}$, rated power, socket ids	NILM, office specific appliance classification, smart meters
MEULPv2 Saldanha et al. 2012	THA HVAC	IL NIL	23	Mains: 1min Plug: 1min	P	N/A	N/A	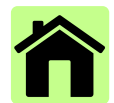	N/A	App$_{\text{brand}}$, App$_{\text{model}}$, socket ids, turn-on delay, house attributes	Demand-size management, building performance simulation
COOLL Picon et al. 2016	THA	IL	N/A	Plug: 100khz	P,I,V	N/A	N/A	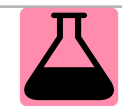	N/A	App$_{\text{brand}}$, App$_{\text{model}}$, rated power, location	NILM, transient analysis
UK-DALE Kelly et al. 2015	THA EH GB	IL NIL	5	Mains: 16kHz Plug: 6s	P,S,V Q, ϕ, E	N/A	N/A	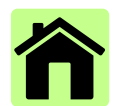	N/A	App$_{\text{brand}}$, App$_{\text{model}}$ On/off transitions, occupancy, house characteristics, heating system, rated power	NILM, grid modeling, demand response
DRED Uttama Nambi et al. 2015	THA	IL NIL	1	Mains: 1Hz Plug: 1Hz	P	T_{in},RH_{in} WiFi,BT	N/A	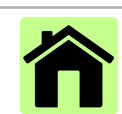	N/A	Occupancy, floor plan App$_{\text{type}}$, App$_{\text{model}}$, rated power, location	Real-time NILM, demand-response
DATA-PORT Parson et al. 2015	THA AC	IL NIL	722	Mains: 1min Plug: 1min	P	N/A	N/A	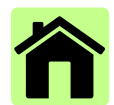		House characteristics, App$_{\text{type}}$, installation date, N° circuits	NILM, behavioral studies demand-response grid modeling
ACS-F2 Ridi et al. 2014	THA	IL	N/A	Plug: 10s	P,Q,S ϕ,V,f,I	N/A	N/A	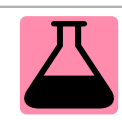	Appliance states - on/off/standby, intermediate states	App$_{\text{type}}$, App$_{\text{model}}$, acquisition protocol, session information	Appliance classification, algorithm benchmark
PLAID Gao et al. 2014	THA	IL	56	Plug: 30kHz	V, I, P P, Q, ϕ, f	N/A	Startup transients, steady-state	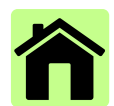	N/A	App$_{\text{type}}$, App$_{\text{model}}$, noise annotations	Appliance fingerprinting, NILM algorithm benchmarking, high-res load analysis

Continued on next page

Supplementary Table 1 (continued).

Dataset/ Year	Devices	Samp. Type	N° houses	Samp. Freq.	Energy Vars	Envir. Vars	Oper. Vars	Envir.	User op. Vars	Metadata	Main Use Cases
GREEND<br>Monacchi et al. 2014	THA	IL<br>NIL	8	Plug: 1Hz	P	T_{in},RH_{in}	Appliance state changes	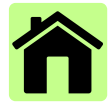	N/A	Appliance types	NILM,<br>occupancy detection,<br>appliance usage mining
ECO<br>Beckel et al. 2014	THA	IL<br>NIL	6	Mains: 1Hz<br>Plug: 1Hz	P,Q,V<br>I,ϕ	N/A	N/A	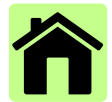	N/A	Appliance types,<br>plug label,<br>household attributes	NILM evaluation,<br>occupancy detection,<br>energy disaggregation
iAWE<br>Batra et al. 2013	THA<br>Water motor	IL<br>NIL	1	Mains: 1Hz<br>Circuits: 20Hz<br>Plug: 1Hz<br>Water: 5Hz	P, Q, S,<br>V, I, f, ϕ	T_{in}, RH_{in}<br>Light, Sound<br>Motion	Appliance states<br>Water pump cycles<br>HVAC usage	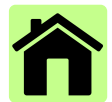	N/A	Appliance metadata<br>Star ratings<br>Circuit mappings<br>Room labels	NILM research<br>Developing country energy studies<br>Water-energy optimization
AMPds<br>Makonin et al. 2013	THA<br>Gas<br>Water	IL	1	Plug: 1min	P, Q, S,<br>V, I, f, ϕ	N/A	Appliance states<br>HVAC cycles<br>Water heater usage	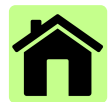	N/A	Appliance types<br>Circuit mappings<br>EnerGuide rating<br>Sub-meter labels	NILM research<br>Eco-feedback systems<br>Multi-utility conservation studies
Smart*<br>Barker et al. 2012	THA<br>PV<br>Wind	IL<br>NIL	3	Mains: 1s<br>Circuits: 1s<br>Plug: 2.5s<br>Renewables: 5s	P, S, V,<br>I, f	$T_{\text{in/out}}$,<br>$RH_{\text{in/out}}$<br>v_{wind}<br>G,P_{atm}	Switch on/off/dim<br>Thermostat states<br>Motion/door events<br>HVAC cycles	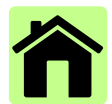	N/A	Circuit mappings<br>Room labels<br>Appliance types<br>Solar/wind specs	NILM benchmarking<br>Demand response<br>Privacy studies
BLUED<br>Filip et al. 2011	THA	IL<br>NIL	1	Mains: 12kHz<br>Plug: 1kHz	P,S,V<br>I,f	N/A	N/A	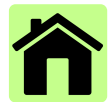	N/A	Appliance types,<br>phase information	Event-based NILM,<br>appliance classification,<br>transient analysis
TRACE-BASE<br>Reinhardt et al. 2012	THA<br>TOA	IL	N/A	Plug: 1s,8s	P	N/A	N/A	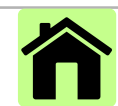<br>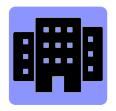	N/A	App_{type}<br>$\text{App}_{\text{model}}$<br>trace metadata	Appliance classification benchmarking
REDD<br>Kolter et al. 2011	THA	IL<br>NIL	5	Mains: 15kHz<br>Circuits: 0.5Hz<br>Plug: 1Hz	P,Q,S<br>V,I, ϕ	N/A	N/A	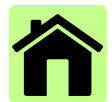	N/A	Circuit labels<br>device categories	Energy disaggregation,<br>NILM research,<br>smart grid applications

Note: WM = Washing Machine, DR = Dryer, THA = Typical Household Appliances, TOA = Typical Office Appliances, HP = Heat Pump, EH = Electric Heater, AC = Air Conditioner, HVAC = Heating, Ventilation, and Air Conditioning. IL = Intrusive Load Monitoring, NIL = Non-Intrusive Load Monitoring. P, Q, S = Real, Reactive, and Apparent Power. V, I = Voltage and Current. ϕ = Phase Angle. f = Frequency. $T_{\text{in/out}}$ = Indoor/Outdoor Temperature. $RH_{\text{in/out}}$ = Relative Humidity (Indoor/Outdoor). P_{atm} = Atmospheric Pressure. P_{water} = Water Pressure. T_{in}, T_{out} = Inlet/Outlet Pipe Temperature. $Q_{\text{w-in}}$, $Q_{\text{w-out}}$ = Inlet/Outlet Water Quantity. a_{vib} = Vibration Acceleration. $T_{\text{w-in}}$, $T_{\text{w-out}}$ = Water Inlet/Outlet Temperature. T_{drum}, T_{door}, $T_{\text{lint-trap}}$ = Drum, Door, and Lint Trap Temperatures. V_{pickup} = Audio Signal (WAV Recording). m_{laundry} = Laundry Weight (Before Wash). m_{wet}, m_{dry} = Laundry Weight After Washing (Wet) and After Drying. P_{wash}, P_{dry} = Power During Washing and Drying. T_{set} = Selected Washing Program Temperature. V_{cond} = Condensed water volume (total condensate collected during operation). App_{brand}, App_{model} = Appliance Brand and Model. Lbl_{heating}, $Lbl_{\text{water-in}}$, $Lbl_{\text{water-out}}$, $Lbl_{\text{centrifuge}}$ = Labels Indicating Specific Cycle Phases (Heating, Water Inlet/Outlet, Centrifuge).

References (Supplementary Information)

Barker, Sean et al. (2012). "Smart*: An open data set and tools for enabling research in sustainable homes". In: *SustKDD, August* 111.112, p. 108.

Batra, Nipun et al. (2013). "It's Different: Insights into home energy consumption in India". In: *Proceedings of the 5th ACM workshop on embedded systems for energy-efficient buildings*, pp. 1–8.

Beckel, Christian et al. (2014). "The ECO data set and the performance of non-intrusive load monitoring algorithms". In: *Proceedings of the 1st ACM conference on embedded systems for energy-efficient buildings*, pp. 80–89.

Filip, Adrian et al. (2011). "Blued: A fully labeled public dataset for event-based nonintrusive load monitoring research". In: *2nd workshop on data mining applications in sustainability (SustKDD)*. Vol. 2012.

Gao, Jingkun et al. (2014). "Plaid: a public dataset of high-resoultion electrical appliance measurements for load identification research: demo abstract". In: *proceedings of the 1st ACM Conference on Embedded Systems for Energy-Efficient Buildings*, pp. 198–199.

Kelly, Jack and William Knottenbelt (2015). "The UK-DALE dataset, domestic appliance-level electricity demand and whole-house demand from five UK homes". In: *Scientific data* 2.1, pp. 1–14.

Kolter, J Zico and Matthew J Johnson (2011). "REDD: A public data set for energy disaggregation research". In: *Workshop on data mining applications in sustainability (SIGKDD), San Diego, CA*. Vol. 25. Citeseer. Citeseer, pp. 59–62.

Kriechbaumer, Thomas and Hans-Arno Jacobsen (2018). "BLOND, a building-level office environment dataset of typical electrical appliances". In: *Scientific data* 5.1, pp. 1–14.

Makonin, Stephen et al. (2013). "AMPds: A public dataset for load disaggregation and eco-feedback research". In: *2013 IEEE electrical power & energy conference*. IEEE, pp. 1–6.

Monacchi, Andrea et al. (2014). "GREEND: An energy consumption dataset of households in Italy and Austria". In: *2014 IEEE International Conference on Smart Grid Communications (SmartGridComm)*. IEEE, pp. 511–516.

Parson, Oliver et al. (2015). "Dataport and NILMTK: A building data set designed for non-intrusive load monitoring". In: *2015 ieee global conference on signal and information processing (globalsip)*. IEEE, pp. 210–214.

Picon, Thomas et al. (2016). "COOLL: Controlled on/off loads library, a public dataset of high-sampled electrical signals for appliance identification". In: *arXiv preprint arXiv:1611.05803*.

Reinhardt, Andreas et al. (2012). "On the accuracy of appliance identification based on distributed load metering data". In: *2012 Sustainable Internet and ICT for Sustainability (SustainIT)*. IEEE, pp. 1–9.

Ridi, Antonio, Christophe Gisler, and Jean Hennebert (2014). "ACS-F2—A new database of appliance consumption signatures". In: *2014 6th international conference of soft computing and pattern recognition (SoCPaR)*. IEEE, pp. 145–150.

Saldanha, Neil and Ian Beausoleil-Morrison (2012). "Measured end-use electric load profiles for 12 Canadian houses at high temporal resolution". In: *Energy and Buildings* 49, pp. 519–530.

Schlemminger, Marlon et al. (2022). "Dataset on electrical single-family house and heat pump load profiles in Germany". In: *Scientific data* 9.1, p. 56.

Uttama Nambi, Akshay SN, Antonio Reyes Lua, and Venkatesha R Prasad (2015). "Loced: Location-aware energy disaggregation framework". In: *Proceedings of the 2nd acm international conference on embedded systems for energy-efficient built environments*, pp. 45–54.

Wenninger, Marc, Andreas Maier, and Jochen Schmidt (2021). "DEDDIAG, a domestic electricity demand dataset of individual appliances in Germany". In: *Scientific Data* 8.1, p. 176.

Yan, Lei et al. (2020). "LIFTED: Household appliance-level load dataset and data compression with lossless coding considering precision". In: *2020 IEEE Power & Energy Society General Meeting (PESGM)*. IEEE, pp. 1–5.
